# Diffuse reflectance spectroscopy to monitor murine colorectal tumor progression and therapeutic response

**DOI:** 10.1117/1.JBO.25.3.035002

**Published:** 2020-03-06

**Authors:** Ariel I. Mundo, Gage. J. Greening, Michael J. Fahr, Lawrence N. Hale, Elizabeth A. Bullard, Narasimhan Rajaram, Timothy J. Muldoon

**Affiliations:** a University of Arkansas, Department of Biomedical Engineering, Fayetteville, Arkansas, United States; b University of Arkansas, Department of Computer Science, Fayetteville, Arkansas, United States; c University of Arkansas, Department of Chemistry and Biochemistry, Fayetteville, Arkansas, United States

**Keywords:** azoxymethane, colon cancer, diffuse reflectance spectroscopy, endoscopy, hemoglobin content, oxygen saturation, metronomic chemotherapy, neoadjuvant chemotherapy, phantom

## Abstract

**Significance:** Many studies in colorectal cancer (CRC) use murine ectopic tumor models to determine response to treatment. However, these models do not replicate the tumor microenvironment of CRC. Physiological information of treatment response derived via diffuse reflectance spectroscopy (DRS) from murine primary CRC tumors provide a better understanding for the development of new drugs and dosing strategies in CRC.

**Aim:** Tumor response to chemotherapy in a primary CRC model was quantified via DRS to extract total hemoglobin content (tHb), oxygen saturation (${\mathrm{StO}}_{2}$), oxyhemoglobin, and deoxyhemoglobin in tissue.

**Approach:** A multimodal DRS and imaging probe (0.78 mm outside diameter) was designed and validated to acquire diffuse spectra longitudinally—via endoscopic guidance—in developing colon tumors under 5-fluoruracil (5-FU) maximum-tolerated (MTD) and metronomic regimens. A filtering algorithm was developed to compensate for positional uncertainty in DRS measurements

**Results:** A maximum increase in ${\mathrm{StO}}_{2}$ was observed in both MTD and metronomic chemotherapy-treated murine primary CRC tumors at week 4 of neoadjuvant chemotherapy, with 21±6% and 17±6% fold changes, respectively. No significant changes were observed in tHb.

**Conclusion:** Our study demonstrates the feasibility of DRS to quantify response to treatment in primary CRC models.

## 1 Introduction

Colorectal cancer (CRC) is the fourth leading cause of cancer death in the world, and in the United States, it accounted for 145,600 new cases in 2019.^1^^,^^2^ Preclinical research aimed at understanding the mechanisms of this disease and developing new therapies, frequently focuses on the use of ectopic xenografts or allografts in murine models to study molecular shifts in tumors.^3^^–^^5^ However, these xenograft/allograft animal models do not properly take into account the influence of the tumor microenvironment^6^^–^^8^ and lack predictive power regarding clinical phase II performance,^9^ which limits the inferences from the data obtained from them. Primary mouse models of CRC, which more closely replicate primary tumors seen in humans—tumors that develop from dysplastic lesions within the colon epithelium itself—resemble the tumor microenvironment and anatomic location, making them better suited for the study of this disease. These models can be derived via carcinogen administration—such as azoxymethane (AOM)—or in transgenic animals (such as ${\mathrm{APC}}^{\min}$).^10^

In particular, these models can be used to study antitumor drugs and dosing strategies for neoadjuvant chemotherapy (NAC), which is administrated clinically before surgical resection with the aim of shrinking the tumor in patients with locally advanced disease (stage II or III).^11^ Typically, the standard NAC regimen is based on the maximum-tolerated dose (MTD) approach,^8^ which requires the cycling of treatment due to its associated toxicity and side effects (nausea, fever, pain). This cycling can have the unintended effect of allowing the tumors to recover in between administration of the antitumor agent. In contrast, the concept of low-dose continuous (metronomic) chemotherapy has been developed over the past 15 years, as it minimizes treatment side effects while targeting the endothelial cells of the tumor vasculature, which are not allowed to recover.^12^^,^^13^ This therapy has been explored as NAC in clinical studies of ovarian, cervical, and breast cancers, where tumor reduction was observed.^14^^–^^16^ Novel administration strategies for CRC—with the intent of understanding the mechanistic effects of this approach, as well as to identify potential clinically viable (endoscopic) biomarkers of positive therapy response—require the use of *in vivo*, primary models of the disease.

While primary CRC tumors may be removed from the mice following euthanasia or biopsied, longitudinal, *in-vivo* studies can track tumor progression (or involution) in response to therapy as well as interaction with the surrounding tissue. A variety of endoscopic imaging modalities that have been developed to study the colon of mice *in vivo* include optical coherence tomography (OCT), laser-induced fluorescence (LIF), label-free multiphoton microscopy (MPM), and Raman spectroscopy.^17^^–^^19^ These emerging imaging techniques have demonstrated that high-resolution imaging of the colon epithelium (OCT, MPM), extraction of fluorescence spectra from tissue (LIF), and spectral shifts to discriminate malignant tissue (Raman) are feasible in the preclinical setting but have not been used to track tumors longitudinally. However, these studies have highlighted the intrinsic challenge of *in-vivo* imaging and spectroscopy within the colonic environment, which complicates reproducibility and accuracy of measurements, making this an important factor that investigators need to consider for the acquisition of endoscopy-derived information in primary CRC models.

In this context, diffuse reflectance spectroscopy (DRS) is a maturing technology that features a minimally invasive, nonionizing optical technique that enables *in-vivo* quantification of tissue oxygen saturation (${\mathrm{StO}}_{2}$), total hemoglobin content (tHb), oxyhemoglobin (${\mathrm{HbO}}_{2}$) and deoxyhemoglobin (Hb). This technique has been applied in clinical studies of breast cancer to determine the response of tumors to NAC^20^^,^^21^ and to longitudinally monitor tumor response to treatment in subcutaneous xenografts.^22^ While DRS offers promising advantages for study of *in-vivo* tumor response to therapy, deployment via an endoscopic approach within murine models faces similar practical challenges as previously described.

Our group has previously used DRS in subcutaneous models of CRC,^23^ demonstrating the feasibility and advantages of extracting *in-vivo* physiological information. However, as the diameter of a mouse colon is below 4 mm and the thickness of the colonic wall ranges between 180 and 800  μm,^6^^,^^7^ the miniaturization of DRS instrumentation presents different challenges: the probe needs to be miniaturized while maintaining an adequate signal-to-noise ratio,^24^ and peristalsis and subject breathing can both cause displacement between the probe and the tissue when data are collected, thereby introducing artifacts in the collected signal.

For a submillimeter diameter endoscopic DRS probe, the use of a pressure sensor to determine “optimal” contact with tissue is unlikely as miniature sensors are interferometry-based^25^ and they are not suited for direct contact with tissue. To overcome this limitation, we have devised an empirical analysis to model changes in DRS-obtained reflectance spectra due to motion or distance artifacts in order to filter the endoscopically acquired DRS data.

This study presents the design, validation, and endoscopic implementation of a small-diameter [0.78 mm outside diameter (OD)] multimodal optical imaging and DRS probe used in an AOM model of CRC to longitudinally quantify the response *in vivo* to different NAC (MTD or metronomic). To the best of our knowledge, this is the first study that compares endoscopically acquired DRS-derived physiological parameters from a model of CRC using different chemotherapy regimens.

## 2 Materials and Methods

### 2.1 Multimodal Optical Imaging and Diffuse Reflectance Spectroscopy Probe Design

**Fig. 1:**
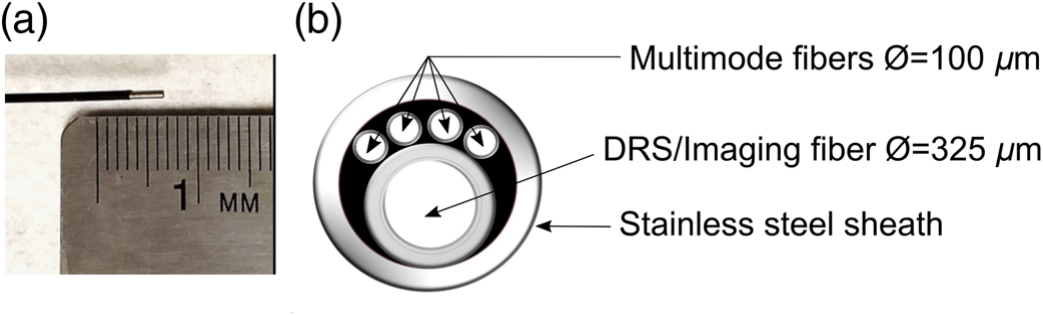
Multimodal DRS probe. (a) Distal end of the probe showing the optical fibers (Ø=100  μm each, polyimide buffer for each fiber in gray) and the DRS/imaging fiber (Ø=325  μm, silicone resin coating in dark gray). (b) Optical and DRS/imaging fiber dimensions and configuration.

A small diameter (0.78 mm OD) multimodal optical imaging and DRS probe (Myriad Fiber Imaging, Dudley, Massachusetts) was designed to be deployed through the biopsy port of a commercial veterinary colonoscope (Karl Storz COLOView). The distal end of the probe comprises four silica optical fibers with a core diameter of 100±3  μm each, individually surrounded by a polyimide buffer; and an imaging/DRS fiber (Fujikura FIGH-10-350S) with an image circle diameter of 325±20  μm, a fiber center-to-center distance of 3  μm, coated with silicon resin [Fig 1(b)]. The center-to-center distance between each individual optical fiber and the imaging fiber is ∼290  μm.

The total length of the probe is 2 m. At the distal end, the fibers are bundled together under a stainless-steel sheath for the first 3 mm [Figs. 1(a) and 1(b)], after which they are under a black polyimide cover for 1 m, finally branching individually for the remaining distance (1 m) each finishing in a SMA095 connector. Using a previously reported microendoscopy system^26^^,^^27^ the imaging component of the probe was characterized (Probe imaging characterization in the Supplementary Material).

### 2.2 Probe Diffuse Reflectance Spectroscopy Characterization: Calibration, Validation, and Sampling Depth

The inverse lookup table (LUT) approach was used for DRS, which has been described elsewhere.^28^^–^^30^ Briefly, liquid (calibration) phantoms with known optical properties ($\mu_{a}$, ${\mu^{\prime }}_{s}$) were created to span a biological relevant range of ${\mu^{\prime }}_{s}$ and $\mu_{a}$ in tissue.^31^^,^^32^ The phantom optical properties, system configuration, and LUT appear in Figs. S2(a), S2(b), S2(e), and S3(a) in the Supplementary Material. Detailed methods appear in DRS Calibration in the Supplementary Material.

For validation, five validation phantoms were created [Figs. S2(c) and S2(d) in the Supplementary Material] and a nonlinear optimization routine was used to extract the optical properties ($\mu_{a}$ and ${\mu^{\prime }}_{s}$) using previously described constraining equations and boundary conditions^23^^,^^28^ and Fig. S3(b) in the Supplementary Material. The methodology for this step appears in DRS Validation in the Supplementary Material.

Sampling depth of the DRS configuration of the multimodal probe was defined as the depth reached by 50% of the photons and calculated as reported elsewhere.^23^^,^^33^ Methods are described in detail in Sampling Depth in the Supplementary Material.

### 2.3 Animal Studies: Azoxymethane Model and Treatments

An AOM model of CRC was used. Nine-week-old female A/J mice (000646 The Jackson Laboratory, Maine, n=23) received AOM injections (A5486, Millipore Sigma) at a dose of 10  mg/kg once a week for 6 weeks. All procedures were approved by the University of Arkansas Institutional Animal Care and Use Committee. Mice were acclimated for 1 week upon arrival, after which the active treatment phase began. During the AOM phase, weight was assessed daily, and subcutaneous sterile saline injections were performed if weight loss was >10% when compared to the weight of the first day of injection.

Mice strain and AOM dosage were based on previously reported criteria and values.^34^ The animals were housed in a 12:12 h light–dark cycle facility and received rodent chow (8640, Teklad) and water *ad libitum*. Wet rodent chow was also provided to the mice during the active AOM treatment phase.

**Fig. 2:**
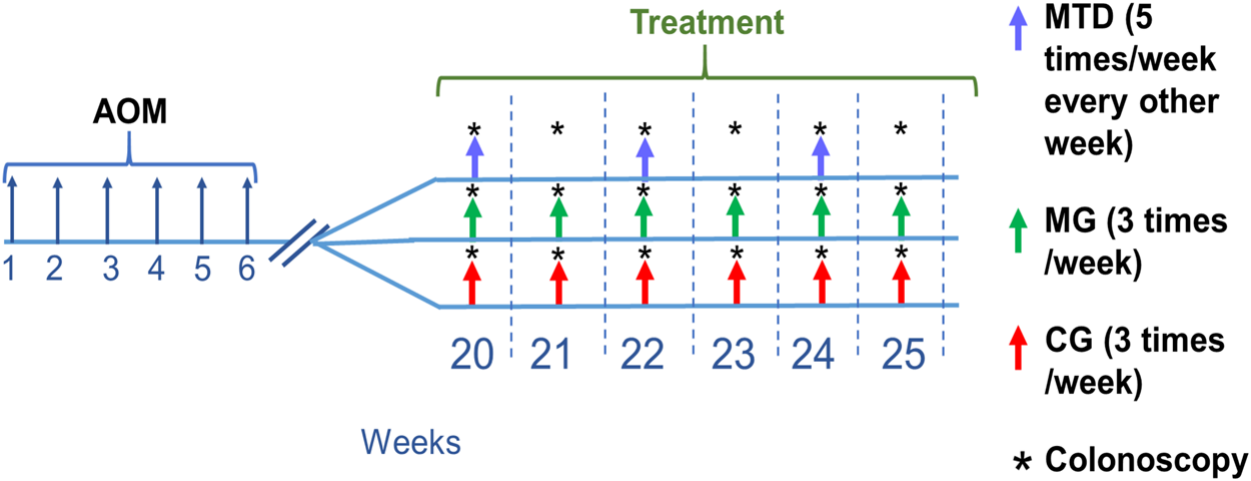
Study timeline. All mice received AOM once a week for 6 weeks at a dose of 10  mg/kg. At 20 weeks post first AOM injection, mice were divided in treatment groups (5-FU NAC or control). MTD mice received 5-FU at a dose of 20  mg/kg (5 times/week every other week), MG mice received 8  mg/kg of 5-FU every other day (3 times/week), and CG mice received 200  μL of sterile saline (3 times/week). Colonoscopies were performed once a week per mouse on the treatment phase, 1 day after the first injection (week 20) and every 7 days subsequently.

At 20 weeks post first AOM injection, the mice started the 6-week treatment phase. Mice were randomly distributed in MTD chemotherapy (n=8), metronomic chemotherapy (MG, n=8), and control (CG, n=7) treatments groups. Chemotherapy was administered 5-FU (Millipore Sigma, F6627). The MTD group received 20  mg/kg of 5-FU intraperitoneally (i.p.) every day for 5 days every other week, and the MG group received 8  mg/kg of 5-FU i.p. every other day three times a week. Control mice received 200  μL of sterile saline i.p. every other day three times a week [Fig. 2]. MTD 5-FU dosage was based on the maximum dose reported by Azrak et al.,^35^ and the break periods followed those seen in NAC in humans. MG dosage (70% reduction) and therapy schedule were based on values reported previously.^36^^,^^37^

### 2.4 Animal Studies: Endoscopic Procedures

**Fig. 3:**
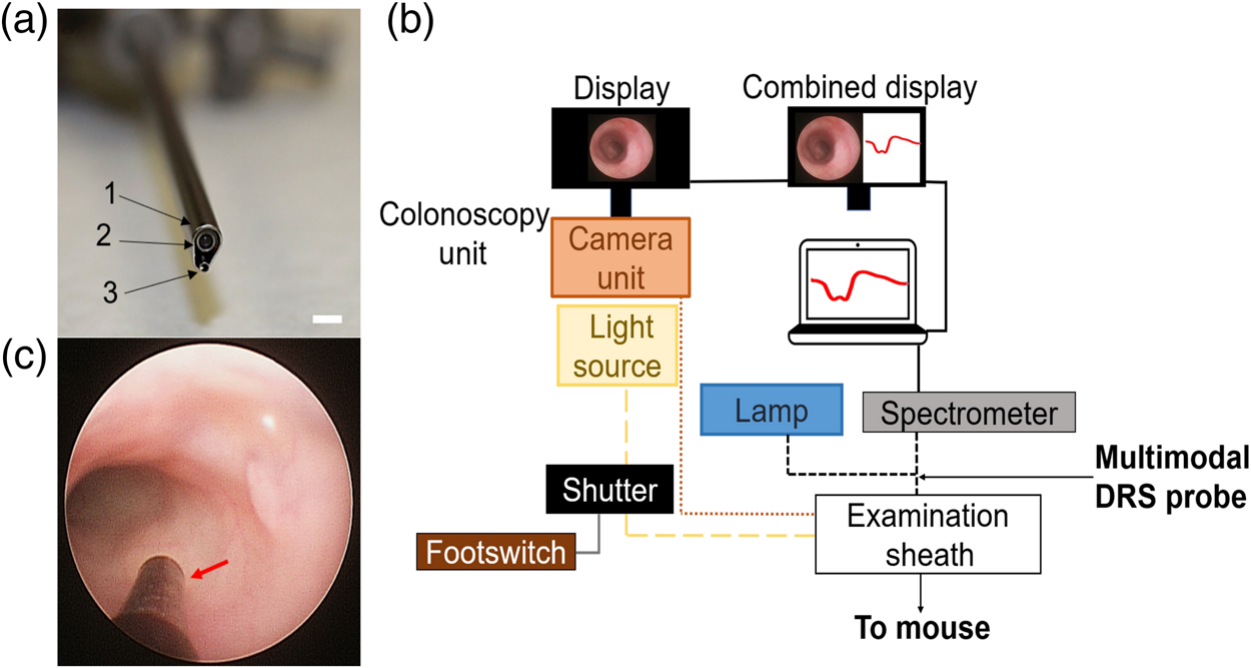
Integrated colonoscopy system setup and *in-vivo* visualization. (a) System configuration used for colonoscopy/DRS acquisition: (1) examination sheath: (2) colonoscopy camera: (3) multimodal DRS probe. Scale bar: 2.3 mm (b) System setup for the colonoscopy/DRS data acquisition. The shutter is activated by a footswitch to block the light from the colonoscopy light source thus enabling only the light of the spectroscopy lamp for DRS measurements. (c) Visualization of the multimodal DRS probe within the colonoscopy camera FOV during the colonoscopy procedure.

To perform integrated DRS and imaging on the endoscopic procedures, a commercial colonoscope unit (Karl Storz COLOView) was used, in a similar manner reported by other groups^38^^,^^39^ but in conjunction with the multimodal DRS probe. To achieve this, the DRS probe was deployed through one of the insufflation ports of the examination sheath [Fig. 3(a)] of the colonoscopy system, thus allowing visual determination of the position of the probe in the colon before acquiring data [Fig. 3(c)]. The configuration for data acquisition for the multimodal DRS probe was the same, as previously indicated in Sec. 2.2 and in DRS Calibration in the Supplementary Material. A footswitch-triggered shutter was implemented to block the light of the colonoscopy system for spectroscopic measurements [Fig. 3(b)]. Colonoscopy, spectroscopy, and combined displays were used to have a holistic view of the procedure.

At 18 weeks post first AOM injection, mice were anesthetized using 2% isoflurane and underwent exploratory imaging-only colonoscopies to assess tumor size. At 20 weeks, the tumors were determined to be roughly the diameter of the DRS probe (1 mm across), and treatment (5-FU or saline) was initiated. For the first week of treatment, colonoscopies where DRS data was collected were performed 24 h after the first injection, and subsequently repeated every 7 days for 6 weeks per mouse [Fig. 2].

Position of the probe with the tumor was determined visually by keeping the tumor within the camera field of view (FOV) and advancing the DRS probe until it touched the tumor. If deformation of the tumor was visible due to the contact of the probe, the procedure was repeated until contact of the probe caused no visible deformation on the tumor. At that point, the footswitch [Fig. 3(b)] was triggered, the light from the colonoscopy unit was blocked, and contact of the DRS probe on the tumor was confirmed if no light from the spectroscopy lamp was seen to be leaking into the environment. A minimum of 10 spectra were collected per tumor at an integration time of 70 ms. Background (noise) and calibration data were collected, as previously described.

At the end of the 6-week period, mice underwent a final colonoscopy where the colon was stained with 1% fluorescein (F6377, Millipore Sigma) and microendoscopy images were acquired using the system configuration described in Fig. S1(b) in the Supplementary Material, and the deployment of the multimodal DRS probe as in Fig. 3(a) using 5 dB gain, 100 ms shutter at a rate of 10 fps using the FlyCapture^®^ software. After the colonoscopy/microendoscopy procedure, mice were euthanized under 4% isoflurane via cervical dislocation. Colons were excised, placed in OCT, and flash-frozen in liquid isopentane and stored at −80°C.

### 2.5 Positional Sensitivity of the Multimodal Diffuse Reflectance Spectroscopy Probe

Although great care was taken when the DRS data were acquired, the inherent moving of the colonic environment (due to peristalsis, mouse breathing, and operator movement) would introduce artifacts in the spectra, and therefore a threshold would be required to filter the data before quantifying physiological values. This issue was addressed in two steps: First, an *ex-vivo* analysis was used to develop an empirical model for the changes in reflectance due to variations in the positioning of the probe relative to the tissue. Later, the changes in reflectance obtained from the *ex-vivo* model were compared to the *in-vivo* data to determine a threshold in reflectance to reject spectra with angular or displacement artifacts.

#### 2.5.1 *Ex-vivo* analysis of the effects of probe contact angle and distance from tissue

The *ex-vivo* analysis was performed to model the changes in reflectance due to positive displacement (when the DRS probe would be away from the tissue), angle of contact between the probe and the tissue, and negative displacement (when the probe deforms the tissue). Since the thickness of mouse colon is <400  μm, the tissue cannot be placed directly over a surface to acquire DRS data as the specular reflection from the material—due to the close proximity of the light source—would cause contamination in the signal.

**Fig. 4:**
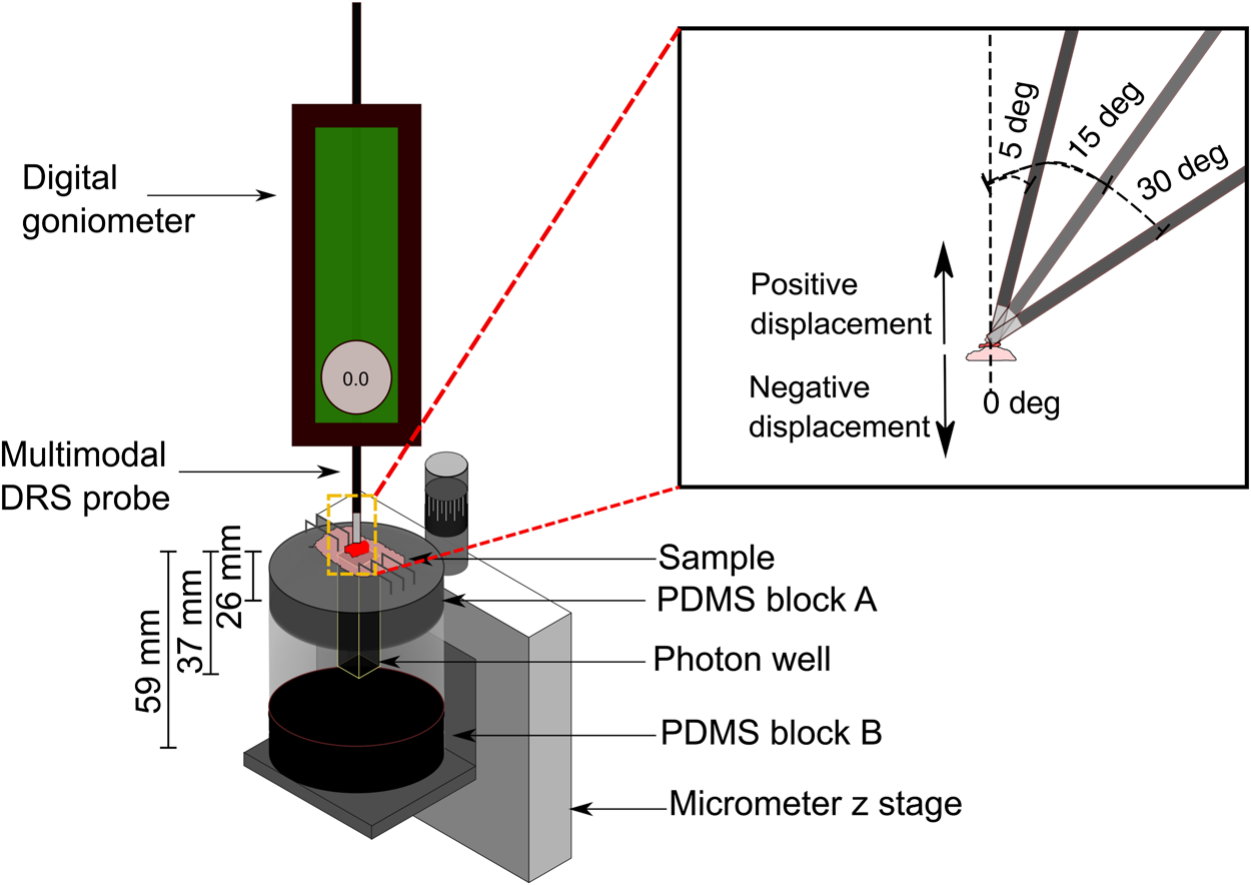
*Ex-vivo* system setup for DRS analysis. Tissue was fixed using staples over a PDMS block that had a lengthwise 1×1  cm section cut and surrounded by black metal foil thus forming a photon well. A micrometer z-stage was used to measure changes in distance in 100  μm increments. Insert presents the variation of the angle of contact between the probe and the tissue for the 0 deg, 5 deg, 15 deg, and 30 deg measurements and the direction of positive and negative displacements between the probe and tissue.

A system was devised [Fig. 4] where the tissue would be placed on top of a block of polydimethylsiloxane (PDMS) with thickness of 26 mm (PDMS block A) and mounted with staples. The block had cut out a 1×1  cm section lengthwise, which had black metal foil surrounding it and extending 11 mm outside of the block (37 mm total length), thus creating a “photon well” that would ensure that no reflected light from the PDMS surface would contaminate the signal. The block was placed on top of a 50-mL beaker with a block made of PDMS and black India ink at the bottom (PDMS block B) that acted as a black body ($\mu_{a}>100\;{\mathrm{cm}}^{-1}$) to absorb any remaining photons that would reach the bottom of the system. The beaker was then placed on top of a micrometer z-stage and the multimodal DRS probe was attached to a digital goniometer fixed to a metal post. In this manner, angle and distance could be precisely tuned.

Freshly resected colons from two mice containing one tumor each were excised from euthanized mice, cut lengthwise to expose the tumors and rinsed with cold phosphate-buffered saline. Tissue was then mounted on the system and the z-stage was moved up until contact between the probe and the tissue was assessed. From there, the z-stage was lowered in 100  μm increments to 1000  μm as this would correspond to a positive displacement of 1.3 probe diameters and would be a plausible upper limit for the distance analysis. The 0 deg, in contact measurements were defined as “optimal” as they would represent the values obtained when no pressure, angle, or distance artifacts are present in the spectra. Spectra were collected per position [Fig. 6(a)] with an integration time of 70 ms with calibration and dark noise spectra acquired accordingly. The process was repeated when the angle was varied at 0 deg, 5 deg, 15 deg, and 30 deg [Fig. 4] to determine changes in reflectance due to changes in contact angle. Finally, negative displacement influence was assessed by placing the probe in contact with the tissue and lifting the z-stage in 100  μm increments up to 700  μm. These measurements were made only with a 0-deg configuration on the system [Fig. 6(b)].

#### 2.5.2 *In-vivo* analysis of optimized probe position

The *ex-vivo* reflectance from colonic tissue was compared to the *in-vivo* reflectance of tumors [Figs. 7(a)–7(c)] where optimal contact was assessed at the moment of spectral acquisition (tumor not deformed by negative displacement, gentle contact, no visible angular separation between the probe) for eight mice (four MTD and four CG). Each mouse had a minimum of five colonoscopies performed, therefore allowing the assessment of temporal changes (longitudinal data) to compare the behavior of *in-vivo* and *ex-vivo* reflectance to determine a threshold to discard data that had pressure, angle, or distance artifacts.

### 2.6 Diffuse Reflectance Spectroscopy Postprocessing: Inverse Lookup Table Model

The filtered *in-vivo* data were used to quantify optical properties and physiological values from tissue (${\mathrm{StO}}_{2}$, tHb, ${\mathrm{HbO}}_{2}$, and Hb) using the inverse LUT model between 475 and 685 nm using constraining equations and boundary conditions as reported elsewhere.^23^^,^^28^ Pigment packaging correction was not introduced in the absorption coefficient as the analysis was performed >400  nm.^29^ Detailed methodology is presented in DRS post-processing (Inverse LUT model) in the Supplementary Material.

### 2.7 Statistical Analysis

The final physiological DRS-derived data were unbalanced with skewness in the distribution of the values (data not shown). The typical repeated measures analysis of variance (rmANOVA) could not be used to analyze the data, as this methodology requires complete observations per subject in order to incorporate it into the analysis and also assumes sphericity for the correlation of the repeated measurements, a condition that may not hold true.^40^

Therefore, a generalized least squares (GLS) method was used to assess the interaction of treatment and time on the DRS physiological data (${\mathrm{StO}}_{2}$, tHb, ${\mathrm{HbO}}_{2}$, and Hb), as this methodology models the correlations between observations in a more flexible way than the rmANOVA model and also supports the use of unbalanced data.^41^ The covariance structure was estimated using an autoregressive process of unequal variances for the observations across time, which was determined to be optimal in base of the log-likelihood value and examination of the correlation of measurements over time. A model where the effect of treatment, time, and their interaction could be assessed over the mean DRS-derived physiological data (${\mathrm{StO}}_{2}$, tHb, ${\mathrm{HbO}}_{2}$, and Hb) was constructed. The significance of fixed effects was set at p<0.05. Statistical analyses and data visualization were performed in R (The R Foundation, version 3.5.2),^42^ using the nlme and ggplot2 packages.^43^^,^^44^

## 3 Results

### 3.1 Validation of the Lookup Table-Inverse Model

**Fig. 5:**
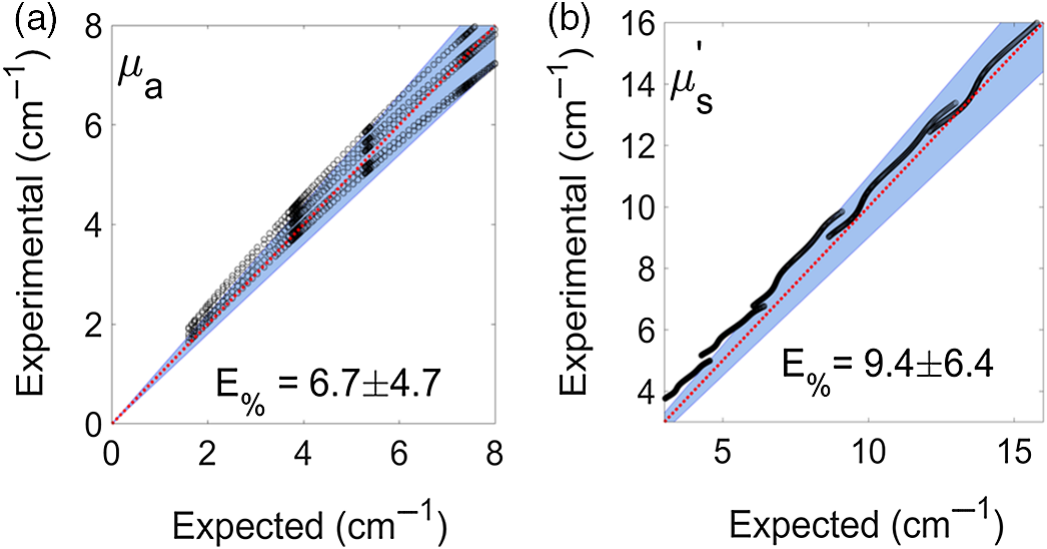
(a) and (b) Percentage errors between the inverse LUT model calculated and the known optical properties of the validation phantoms. Both $\mu_{a}$ and ${\mu^{\prime }}_{s}$ were below 10%. Red line represents perfect agreement between the measures, black dots represent calculated optical properties per phantom, and blue background represents a 10% error margin.

The percentage errors between the phantom optical properties extracted with the inverse LUT model and the known optical properties were quantified as a validation step [Fig. 2(b)]. Average errors were 9.4 and 6.7 for ${\mu^{\prime }}_{s}$ and $\mu_{a}$, respectively [Figs. 5(a) and 5(b)].

### 3.2 *Ex-Vivo* Changes in Reflectance: Variations Due to Positive or Negative Displacement

**Fig. 6:**
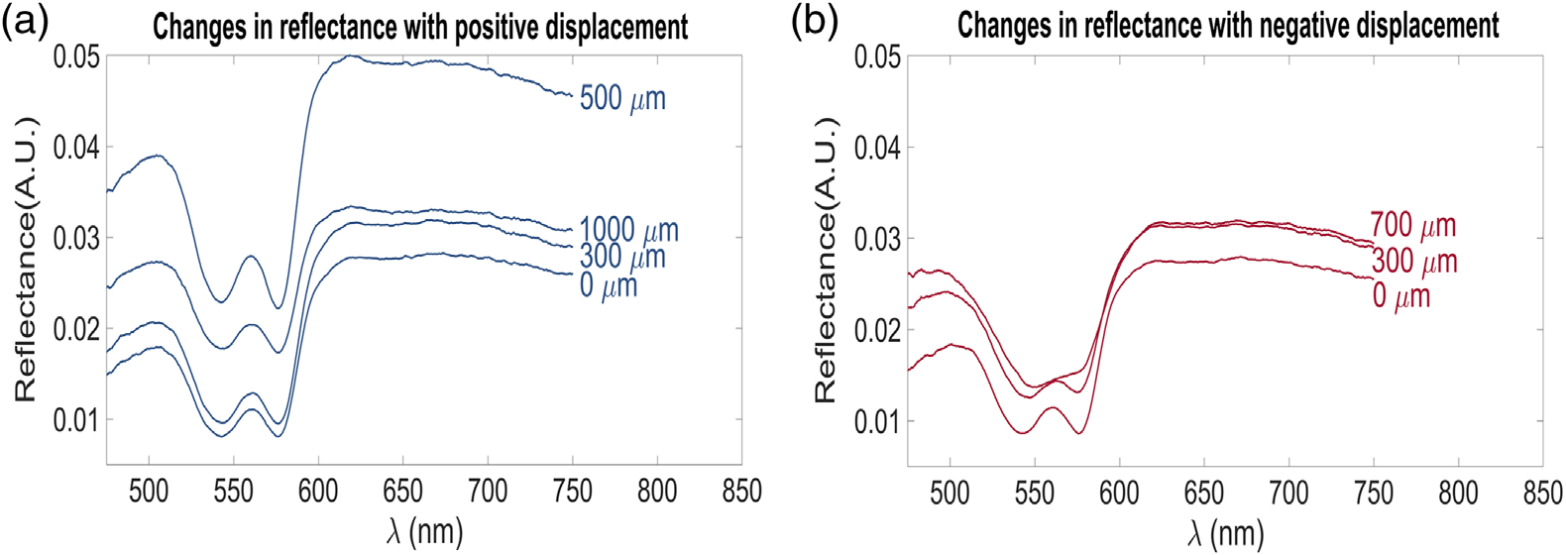
(a) Representative changes in reflectance with positive displacement between the probe and the tissue. Labels per spectra correspond to the distance between the probe and the tissue according to the micrometer scale. (b) Representative changes in reflectance due to negative displacement between the probe and the tissue. Labels per spectra correspond to the distance advanced by the probe over the tissue, as recorded by the micrometer scale.

The *ex-vivo* analysis revealed a minimal increase in reflectance for the first 300 μm of positive displacement, reaching a maximum at a distance of 500  μm and decreasing again in the 600 to 1000  μm [Fig. 6(a)].

For negative displacement, spectra transitioned in the 500 to 600 nm range from a predominantly ${\mathrm{HbO}}_{2}$ spectra to an Hb spectrum [Fig. 6(b)]. The complete set of spectra appears in Figs. S5(a) and S5(b) in the Supplementary Material.

### 3.3 *Ex-Vivo* and *In-Vivo* Optimal Reflectance: *In-Vivo* Reflectance Is Stable and Correlates with Reflectance Acquired *Ex Vivo*

**Fig. 7:**
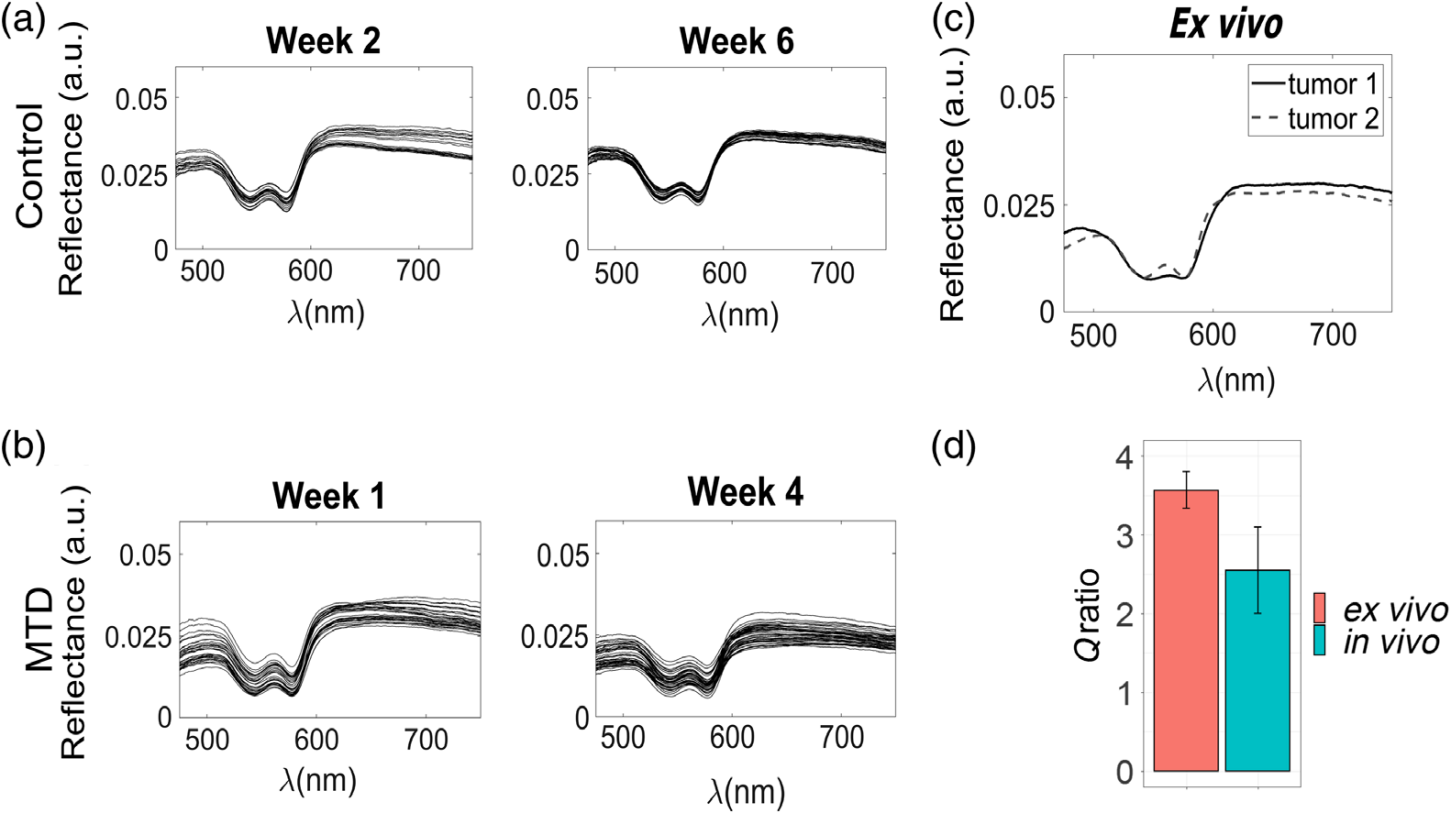
Representative optimal longitudinal reflectance and $Q_{\text{ratio}}$ values. (a) *In-vivo* optimal reflectance—defined as tumor not deformed by negative displacement, gentle contact, no visible angular separation between the probe—acquired for the first day of the indicated week for weeks 2 and 6 for CG mouse. (b) *In-vivo* optimal reflectance for weeks 1, and 4 for treated (MTD) mouse. (c) *Ex-vivo* reflectance from tumors from CG (tumor 1) and MG (tumor 2) mice. Behavior of the spectra was longitudinally consistent *in vivo* and with the *ex vivo* reflectance, thus providing support to the use of a ratio to filter outliers. (d) $Q_{\text{ratio}}$ calculated for optimally acquired data *in vivo* (gentle contact and no separation visible) and *ex vivo* (0 deg, in contact). Bars are means; error bars represent ±1 SD.

Measurements obtained *in vivo* demonstrated that reflectance (R) remained repeatedly below 0.05 at 475 nm [Figs. 7(a) and 7(b)], in line with the values from the optimally acquired *ex-vivo* DRS data [Fig. 7(c)]. The Q bands of hemoglobin would be clearly defined and the region above 600 nm would have a similar linear profile.

### 3.4 *Q*_ratio_ as a Metric to Filter Endoscopically Acquired Diffuse Reflectance Spectroscopy Data

The results from Secs. 3.2 and 3.3 were used to calculate a ratio between the scattering region ($\lambda_{S}=630\;\mathrm{nm}$) and the second Q band of hemoglobin ($\lambda_{Q}=575\;\mathrm{nm}$) per each optimal *ex-vivo* spectra, defined as

$$Q_{\text{ratio}}(i)=[\frac{R_{\lambda s(i)}}{R_{\lambda Q(i)}}],$$ (1)

where i denotes each recorded reflectance in the 475 to 685 nm range and $R_{\lambda s(i)}$, $R_{\lambda Q(i)}$ are the values of the i’th reflectance at 630 and 575 nm, respectively. The $Q_{\text{ratio}}$ was calculated for all the *ex-vivo* spectra with different displacement/angular variations, which demonstrated that changes in angle/displacement resulted in different $Q_{\text{ratio}}s$, whereas values for the 0 deg and 0 to 300  μm positive displacement configuration were similar in both tumors [Fig. S6 in the Supplementary Material]. The $Q_{\text{ratio}}$ for 25 *ex-vivo* optimal spectra were calculated and demonstrated a mean of 3.56±0.23, whereas the $Q_{\text{ratio}}$ (from 258 spectra) was calculated from *in-vivo* data, with a mean value 2.55±0.55 [Fig. 7(d)]. The similarity between the *ex-vivo* and *in-vivo* $Q_{\text{ratio}}s$ supported the use of this metric to differentiate optimal data from data with displacement or angle issues.

Therefore, a filtering algorithm was constructed to discard data that had angular or displacement (positive or negative) artifacts to avoid biasing the DRS data to be analyzed. Detailed description of the algorithm and the corresponding flowchart are summarized in the Filtering Algorithm and Fig. S6 in the Supplementary Material].

### 3.5 Treatment Outcomes: Endoscopic and Microendoscopic Comparisons

**Fig. 8:**
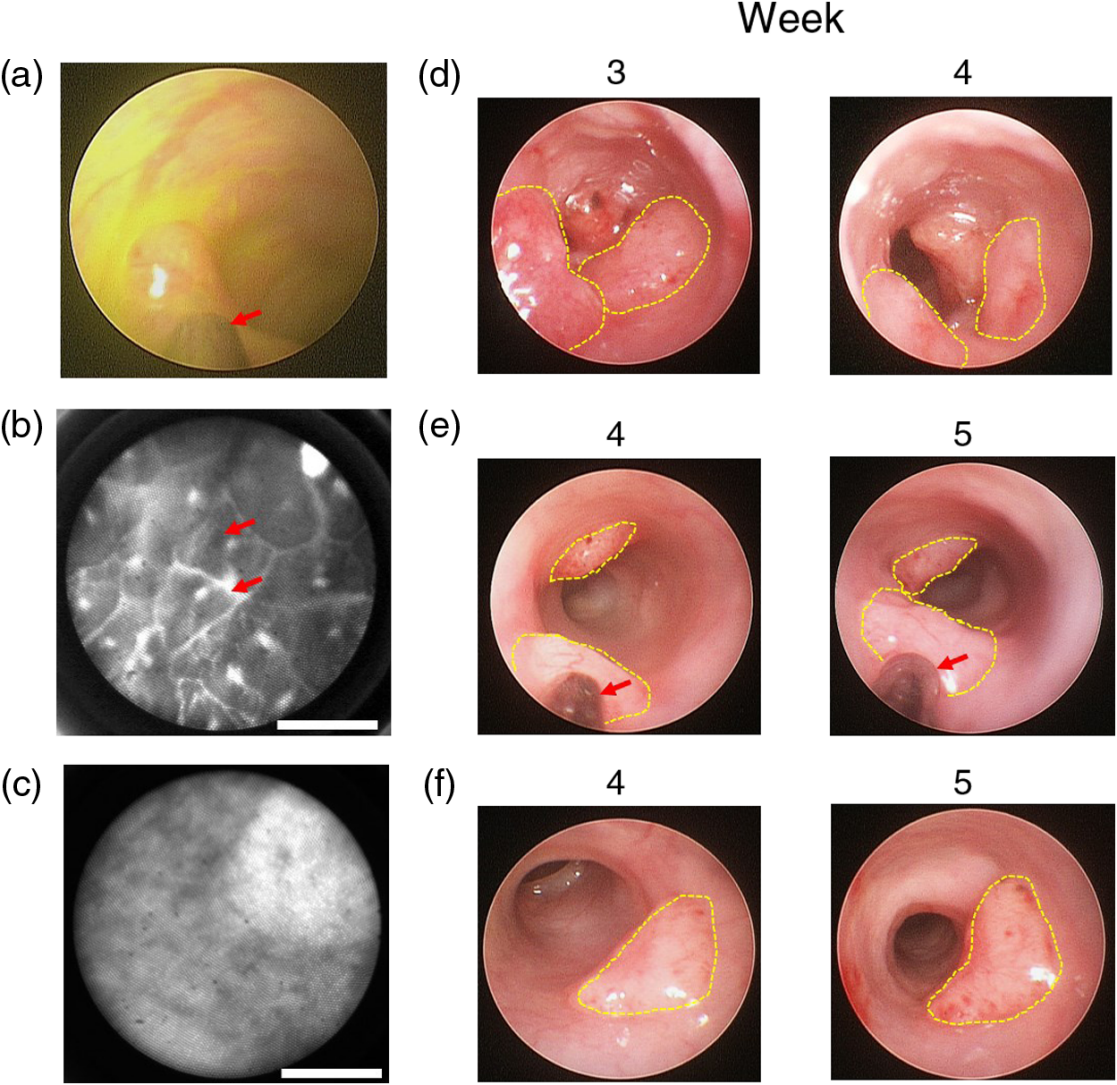
Representative longitudinal endoscopic and microendoscopic imaging of AOM-induced tumors. (a) Endoscopic image of fluorescein stain *in vivo*, where red arrow indicates the multimodal DRS probe. (b) Normal colon imaged using the microendoscopy configuration. Arrows indicate colonic crypts. Scale bar: 100  μm. (c) AOM-induced tumor imaged using the microendoscopy configuration. Scale bar 100  μm. (d) Representative tumors of the MTD group. Regression of the tumor is visible between weeks 3 and 4. (e) Representative tumors of the MG group. (f) Representative tumors of the CG group. In (e) and (f) the size of the tumor increases over time. Tumor vasculature is readily visible in the tumors across all groups. All pictures have been enhanced to emphasize tumor and colonic morphology, yellow dashed lines in (d)–(f) outline the visible volume of the tumor, red arrows indicate the multimodal DRS probe.

Tumors were endoscopically monitored over time during the treatment (chemotherapy or saline) phase [Fig. 2]. Tumor regression was observed in the majority of the MTD-treated tumors between the treatment and nontreatment weeks [Fig. 8(d)]. No tumor regression was observed in the MG-treated tumors and CG tumors [Figs. 8(e) and 8(f)]. Vasculature was visible on the surface of all tumors.

### 3.6 Diffuse Reflectance Spectroscopy-Derived Physiological Parameters from Tumors

**Fig. 9:**
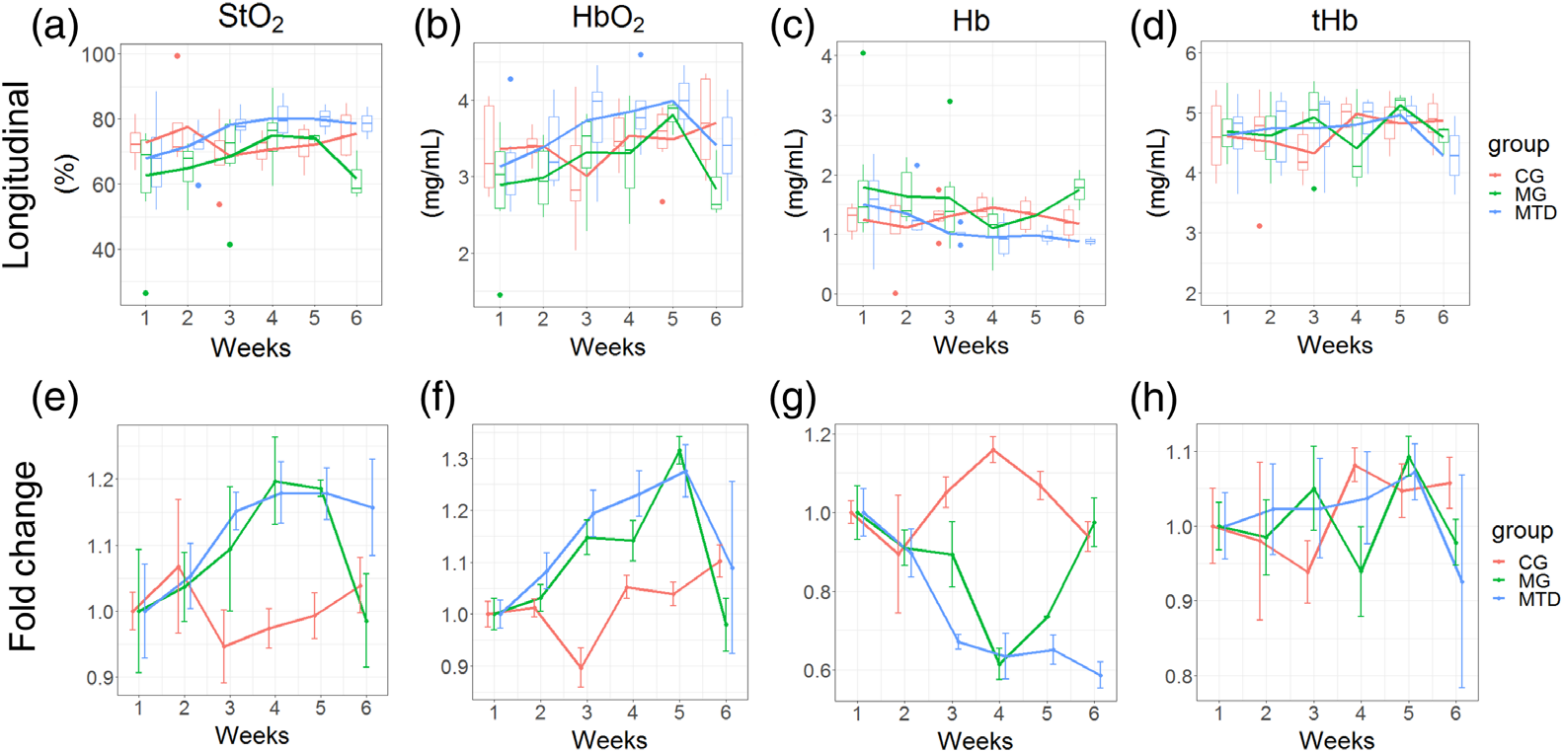
Longitudinal physiological DRS-derived values for tumors under MTD (n=3 to 8), MG (n=2 to 8), and CG (n=3 to 7) groups. (a)–(d) Longitudinal changes in ${\mathrm{StO}}_{2}$, ${\mathrm{HbO}}_{2}$, Hb, and tHb for all groups. Each line represents the physiological data extracted using the inverse LUT model per group per day; boxplots represent the Q1 to Q3 interquartile range per group per day; central line of boxplot is the median value. Points represent outliers. (e)–(h) Longitudinal fold changes in ${\mathrm{StO}}_{2}$, ${\mathrm{HbO}}_{2}$, Hb, and tHb compared to the value from week 1 for each group; error bars represent ±SE/baseline value. Longitudinal trends per mouse and group for ${\mathrm{StO}}_{2}$ and tHb appear in Fig. S8 in Supplementary Material.

Using the inverse LUT model, physiological parameters (${\mathrm{StO}}_{2}$, tHb, ${\mathrm{HbO}}_{2}$, and Hb) were quantified from the AOM-induced tumors. For ${\mathrm{StO}}_{2}$, longitudinal mean values ranged between 66% to 77% (CG), 63% to 76% (MG) and 71% to 86% (MTD) [Fig. 9(a)]. Mean ${\mathrm{StO}}_{2}$ increase was observed in the NAC groups, where the maximum fold change was observed at week 4 (17% for MG and 21% for MTD) [Fig. 9(e)].

For ${\mathrm{HbO}}_{2}$, mean values ranged between 2.7 to 3.7  mg/mL (CG), 2.8 to 3.6  mg/mL (MG), and 3.4 to 4.1  mg/mL (MTD) [Fig. 9(b)]. The maximum fold change was 20% (MTD and MG) at weeks 4 and 5, respectively [Fig. 9(f)]. For Hb, mean values ranged between 1.0 to 1.5  mg/mL (CG), 0.9 to 1.7  mg/mL (MG), and 0.6 to 1.4  mg/mL (MTD) [Fig. 9(c)]. A decreasing trend of Hb was observed in the MTD and MG groups, reaching a 40% and 54% reduction at week 4 [Fig. 9(g)].

Across all groups, tHb values ranged between 4.1 to 5.0  mg/mL (CG), 4.3 to 5.0  mg/mL (MG), and 4.3 to 4.8  mg/mL (MTD) [Fig. 9(d)]. No specific trend in the change of tHb was observed [Fig. 9(h)].

For ${\mathrm{StO}}_{2}$, the interaction of chemotherapy (MTD or MG) and the second-degree polynomial term of time was significant (p=0.0071 and p=0.049, respectively). For Hb, interaction of MG and the second-degree polynomial term was significant (p=0.007). In the case of ${\mathrm{HbO}}_{2}$, interactions between chemotherapy (MTD or MG) and the second-degree polynomial term were significant (p=0.0457 and p=0.036, respectively). For tHb, the interaction of MG and the fifth-degree polynomial term was significant (p=0.0078).

## 4 Discussion

Tumor response to therapy is an important step that needs to be addressed in the development of new drugs, dosing schemes, and treatment strategies that can be translated to the clinic. In this study, DRS was used to extract physiological data from tumors responding to therapy administration *in vivo*, and the inverse LUT model was used due to the scale of the system.^45^ Mean percentage errors for ${\mu^{\prime }}_{s}$ and $\mu_{a}$ were 9.4% and 6.7%, respectively [Figs. 5(a) and 5(b)], in line with values reported in the literature.^46^^–^^48^ The sampling depth for DRS was 263±7  μm at 542 nm.

Diffuse spectra were acquired in developing colon tumors treated with 5-FU using MTD and metronomic regimens. However, as the previously mentioned endoscopic imaging studies have highlighted, variability in the endoscopically acquired signal due to distance from the tissue surface (positive or negative) and angular position between the probe and the tissue are factors that need to be properly controlled in order to accurately quantify tissue optical properties (and extracted physiological data). The orthogonal subtraction method is a statistical approach that accounts for this type of artifact introduced in the signal, but its application in this context is limited by the amount of data required to construct the orthogonal matrix, and because the assumptions required for this methodology may not hold true due to the scale of the system used in this study.^49^

Changes in reflectance due to positional probe variations have been analyzed previously, but the results from these studies are mixed, ranging from increase in reflectance due to pressure^50^ to a decrease in the signal due to the same reason.^51^ Also, datasets are sometimes incomplete, as indicated by Cugmas et al.,^52^ thereby complicating inferences from those studies that could be applicable to our system. Therefore, an empirical model based on the fact that the optimally acquired $Q_{\text{ratio}}$ *ex vivo* (3.56±0.23) was similar to the *in vivo* observed value for optimally acquired spectra (2.55±0.55), [Fig. 7(d)] served as the basis for the use of this metric within an algorithm to automatically filter the data obtained by our submillimeter DRS probe. This methodology allowed us to obtain results specific to our system geometry and tissue of interest. Furthermore, the previously reported use of ratios in reflectance to discriminate between tissues and grade malignancies in the oral mucosa^53^^,^^54^ provided support to the rationale used to filter representative spectra and data with angular or distance artifacts.

In this study, we hypothesize that the observed variations in reflectance due to positive displacement of the probe [Figs. 6(a) and S5(a) in the Supplementary Material] can be explained by the liquid meniscus between the tip of the probe and the tissue for the first 300  μm, which stabilizes the signal. When the liquid meniscus and the tip of the probe separate (distance >300  μm), a sharp increase is observed due to specular reflection caused by the surface of the liquid layer. Finally, as the probe moves farther away from tissue, the reflectance decreases as less light reaches the collecting fiber. During negative displacement, reflectance increased, but on a smaller scale when compared to the previously mentioned positive distance-induced changes. However, in the 500- to 600-nm range—the region where hemoglobin absorption occurs—an increased flattening of the Q bands was seen with incremental negative distance, a behavior previously reported by Popov et al.^55^ and Ti and Lin,^56^ which is attributed to blood displacement and deoxygenation.

For the DRS-extracted physiological values, the observed ${\mathrm{StO}}_{2}$ during the first week of measurements ranged between 65% and 76% across all groups [Fig. 9(a)], in contrast with values extracted from subcutaneous models, where values below 50% have been reported.^23^^,^^57^ We hypothesize that the observed difference can be partially explained by the size of the malignancy, as primary CRC tumors are much smaller (diameter ∼1  mm) than their subcutaneous counterparts, which accordingly would be more hypoxic due to changes in perfusion and necrosis as a result to tumor growth.^58^ But it is also possible that higher ${\mathrm{StO}}_{2}$ values are caused by the blood present on the readily visible superficial vasculature in all groups [Fig. 8], which can be quantified in future studies to address this possibility.

In the NAC groups (MTD or metronomic), an increasing pattern over time was observed in both groups for ${\mathrm{StO}}_{2}$ and ${\mathrm{HbO}}_{2}$ [Figs. 9(a) and 9(b)], coupled with a decrease in Hb and no trend for tHb [Figs. 9(c) and 9(d)]. Chemotherapy (MTD or metronomic) and its interaction with the second-degree polynomial term were significant for ${\mathrm{StO}}_{2}$ and ${\mathrm{HbO}}_{2}$ (p=0.0071 and p=0.049 for ${\mathrm{StO}}_{2}$, p=0.0457 and p=0.036 for ${\mathrm{HbO}}_{2}$, respectively), which indicate that the trends do not follow a linear pattern. Finally, the interaction of MG and the second-degree polynomial term was significant for Hb (p=0.007). While the sample size presents a limitation in this study, it is interesting to note that both NAC treatments cause similar changes in ${\mathrm{StO}}_{2}$, ${\mathrm{HbO}}_{2}$, and Hb over time. Based on our findings, we speculate that the observed changes are possibly caused by a reduced oxygen demand due to necrosis, which would explain the reported increase in ${\mathrm{StO}}_{2}$ and ${\mathrm{HbO}}_{2}$, whereas tHb remained constant. This question will be formally addressed in future studies.

Moreover, our results are in line with previously reported longitudinal trends in the change of ${\mathrm{StO}}_{2}$ and ${\mathrm{HbO}}_{2}$ due to MTD chemotherapy in subcutaneous models.^22^^,^^59^ For the metronomic regimen, the lack of a trend for tHb (indicating no significant treatment effect) and the increase in ${\mathrm{StO}}_{2}$ due to treatment suggest that the treatment did not affect the vascular network of the tumors, as suggested by Fioravanti et al.^36^ In contrast to the conclusions of that study, our results suggest that a 70% reduction on the MTD dose of 5-FU is still capable of producing similar physiological changes (${\mathrm{StO}}_{2}$, ${\mathrm{HbO}}_{2}$, and Hb) as in the MTD regimen due to the fold values observed, although it is not capable of producing tumor regression, which was observed visually (endoscopically) in the MTD regimen [Figs. 8(d) and 8(e)]. The GLS approach^41^ was chosen since the data acquired were unbalanced, and the sample size limits the assumption of a spherical variance–covariance matrix (as required in rmANOVA).^40^ Moreover, the GLS method was preferred as a linear-mixed effects model approach showed that a random effect of subject variability over time was not significant (data not shown).

Our results demonstrate the feasibility and associated challenges in the use of DRS to track the response to treatment in a primary CRC mouse model. To the best of our knowledge, there are limited longitudinal preclinical studies for NAC in this context that monitor tumor hemodynamics. This is the first study that compares physiological information from endoscopically acquired longitudinal DRS data from different NAC strategies. Owing to the ongoing interest in metronomic NAC and the possibility of using this type of therapy as an alternative as NAC in CRC, this study serves as an initial step toward the refining and characterization of this therapeutic approach.

Limitations to the present study include the sample size per group, which limits the size effect observed between treatment groups, and the size of the tumors in the model used (∼1  mm diameter), which are smaller than their human counterparts (∼44  mm diameter)^60^ as the microenvironment landscape is expected to be different, particularly in regard to oxygenation. Also, the model selected is chemically induced and presents different metabolic activity when compared to human tumors.^61^ Finally, the selected metronomic dosage and schedule is also a limitation as changes in the frequency of the treatment or fraction of reduction of the dose can potentially correlate with different physiological values.

## 5 Conclusion

This study outlines the calibration, validation, and implementation of a multimodal DRS probe in a primary CRC murine model with different NAC regimens. Optical errors for the DRS system were comparable to those reported in the literature. *In-vivo* DRS-derived physiological values were computed longitudinally for each group, where NAC (metronomic or maximum-tolerated) caused an increase in ${\mathrm{StO}}_{2}$ and ${\mathrm{HbO}}_{2}$ and a decrease in Hb in tumors, whereas no significant changes in tHb were observed. These results provide an initial step in understanding and quantifying tumor response in different chemotherapeutic regimens. The developed system enables the acquisition of *in-vivo* DRS information in a primary CRC murine model, which could potentially be used to determine tumor response to treatment and guide the development of new drugs for CRC. The concept of a deployable DRS probe could also be possibly translated to the study of other types of cancer that are diagnosed and studied via endoscopy, such as biliary, esophageal, or stomach cancer.
