# Diffuse reflectance spectroscopy to monitor murine colorectal tumor progression and therapeutic response (Erratum)

**DOI:** 10.1117/1.JBO.25.4.049803

**Published:** 2020-04-22

**Authors:** Ariel I. Mundo, Gage. J. Greening, Michael J. Fahr, Lawrence N. Hale, Elizabeth A. Bullard, Narasimhan Rajaram, Timothy J. Muldoon

**Affiliations:** a University of Arkansas, Department of Biomedical Engineering, Fayetteville, Arkansas, United States; b University of Arkansas, Department of Computer Science, Fayetteville, Arkansas, United States; c University of Arkansas, Department of Chemistry and Biochemistry, Fayetteville, Arkansas, United States

## Abstract

The erratum notes a correction to Fig. 5 for the published article.

This article [*J. Biomed. Opt.* **25**(3), 035002 (2019) doi: 10.1117/1.JBO.25.3.035002] was originally published online on 6 March 2020 with an error in Figure 5, corresponding to percentage errors for the optical properties.

The article included a version of the figure that listed the wrong values. The text of Section 3.1 correctly states, “Average errors were 9.4 and 6.7 for $\mu_{s}^{\prime }$ and $\mu_{a}$, respectively [Figs. 5(a) and 5(b)]”; however, in Fig 5 the average errors were incorrectly listed as 6.9 and 5.0. The incorrect figure has been replaced with a corrected version.

Original figure:

**Figure:**
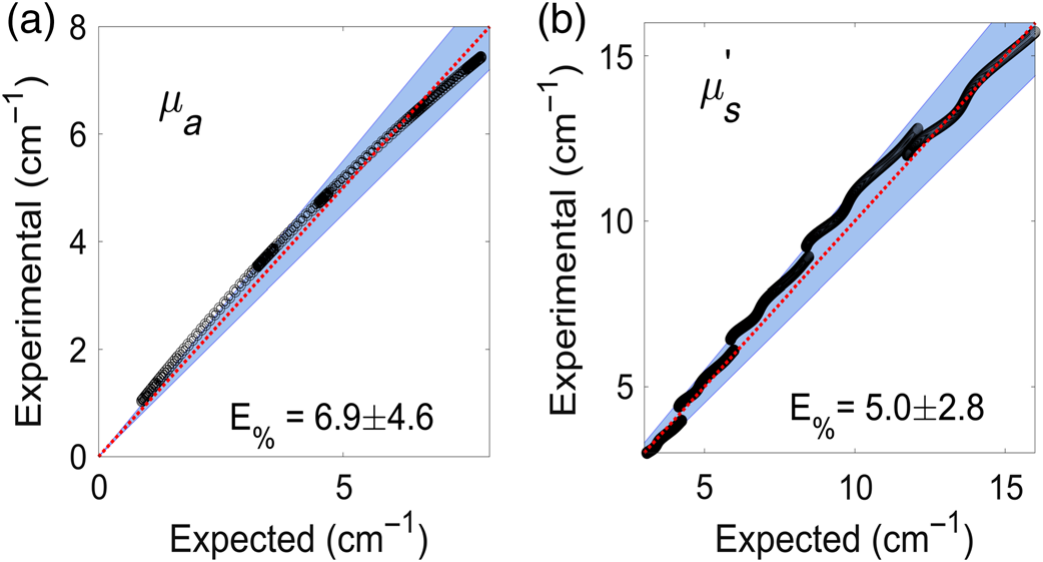


Corrected figure:

**Figure:**
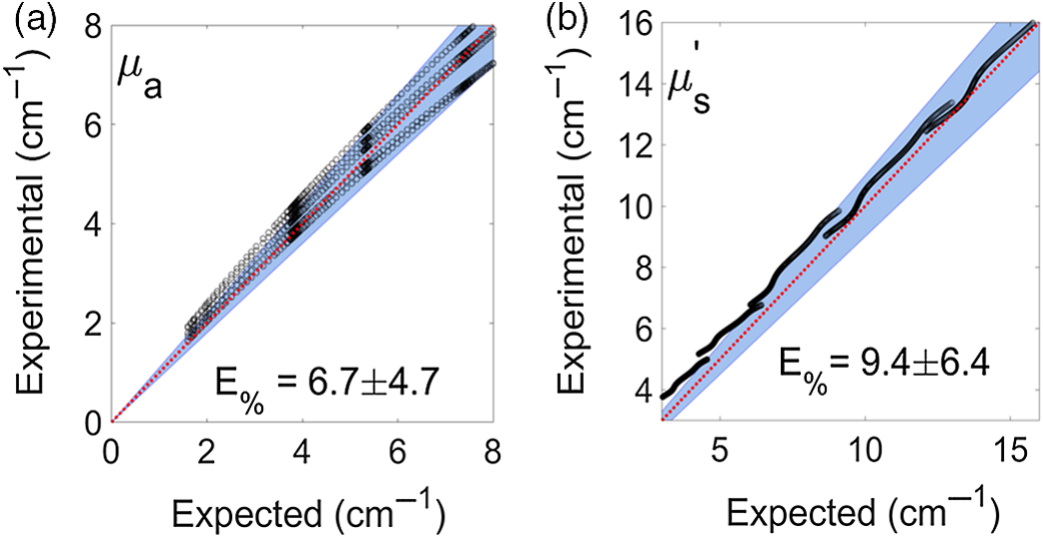


The caption for the figure is unchanged. The results reported were not affected by the error.

This article was corrected online on 27 March 2020.

