# Supplementary material for: Diffuse reflectance spectroscopy to monitor murine colorectal tumor progression and therapeutic response
Source: J Biomed Opt. 2020 Mar 6;25(3):035002. doi: 10.1117/1.JBO.25.3.035002 (PMC7058691; doi:10.1117/1.JBO.25.3.035002)
Supplement: Supplementary file 1 [file JBO_025_035002_SD001.docx]

Diffuse Reflectance Spectroscopy to monitor murine colorectal tumor progression and therapeutic response

Supplemental Materials

**Ariel I. Mundo^1^, Gage. J. Greening^1^, Michael J. Fahr Jr^2^., Lawrence N. Hale^3^, Elizabeth A. Bullard^1^, Narasimhan Rajaram^1^, Timothy J. Muldoon^1^**

^1^Department of Biomedical Engineering

^2^Department of Computer Science

^3^Department of Chemistry and Biochemistry

University of Arkansas

1 University of Arkansas

Fayetteville, AR 72701

**SUPPLEMENTAL METHODS**

## **Probe imaging characterization**

To acquire imaging data, the DRS/imaging fiber was coupled by its proximal (common) end using the SMA connector to a microendoscope system (Fig. S1(b)]. Briefly, the system features a blue LED light (455 center wavelength, Philips, USA), a filter set consisting of 525nm/40nm emission bandpass filter and a 460nm shortpass excitation filter (Chroma Tech, USA), a camera (Flea3(USB) 3.0, FL3-U3-32S2M-CS, FLIR Systems), a 60 mm tube lens and a 10x objective (0.25 NA, Olympus, Japan). Images were acquired with a 5 dB gain, 20 ms shutter at a rate of 48 fps using the FlyCapture® software. The optical specifications for this configuration were determined by using a positive 1951 USAF target at group 5/element 2, which has a width of 13.92 µm/line [Fig. S1(a)]. The determined optical characteristics of the configuration -magnification, %FOV, sampling frequency, etc.- are listed in Table S1.


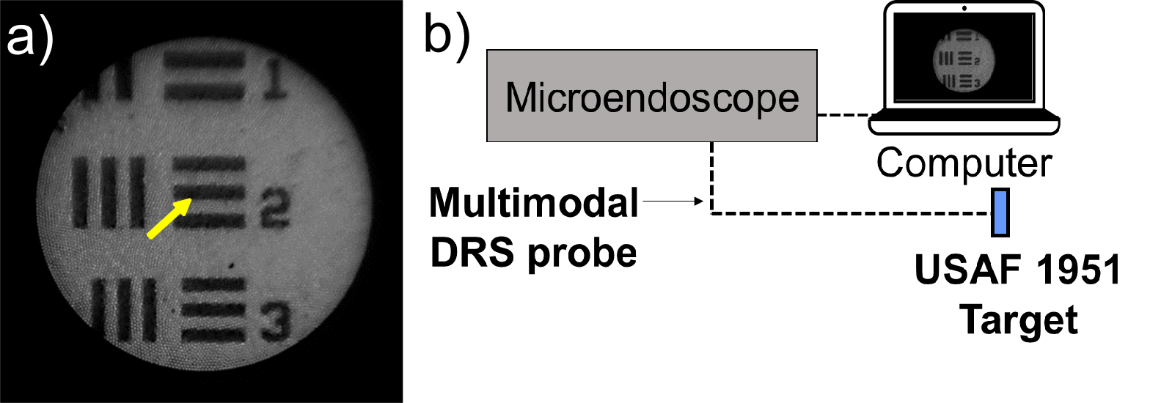


**
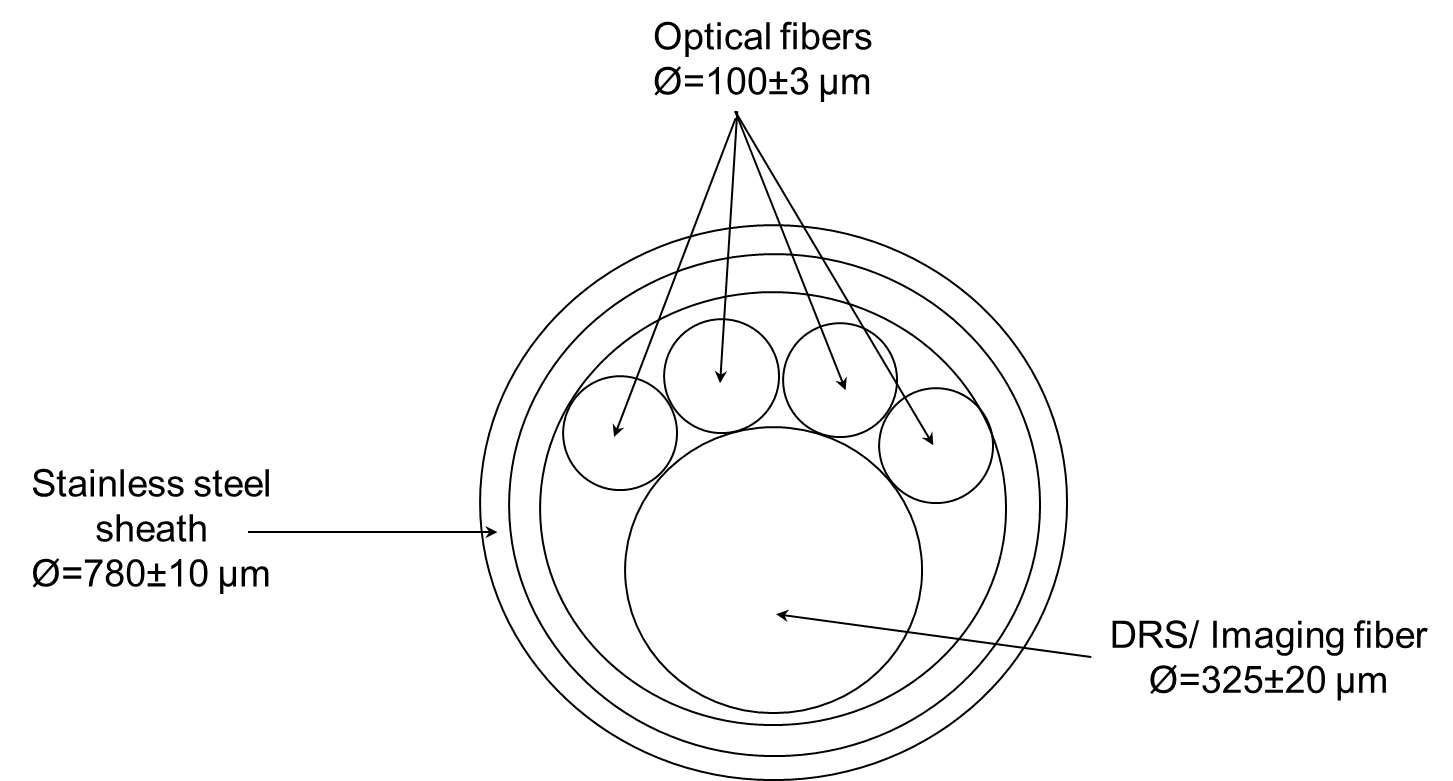
Fig. S1**. a) 1951 USAF Target used to determine the resolution of the imaging fiber, at group 5/element 2 (13.92 µm/line). b) Microendoscopy system configuration.

**Table S1**

Specifications for the imaging system (Microendoscope and imaging fiber of the multimodal DRS probe)

| Quantity | Value |
| --- | --- |
| Spatial resolution (µm) | 3.3 |
| Image sensor (pixels/ µm) | 2.94 |
| Magnification | 7.4 |
| Percent of maximum FOV | 37.4% |
| Sampling frequency (pixels/fiber) | 9.7 |

***DRS Calibration***

To acquire spectroscopic data, one of the optical fibers from the probe was coupled to a broadband tungsten-halogen lamp (HL-2000-HP, Ocean Optics, FL, USA) and the imaging fiber was connected to spectrometer (Flame-T, Ocean Optics, FL, USA) [Fig. S2(e)]. A set of 12 liquid (calibration) phantoms with known optical properties (μ_a_, μ’_s_) were created using polystyrene beads (Polybead® 07310-15, diameter= 1μm, PolySciences, PA) as the scattering agent, and a mixture of red and blue food dyes (McCormick, MD) as the absorbing agent and demineralized water as the solvent. The absorption coefficient for the food dye mix was determined by the Beer-Lambert law using a spectrophotometer (ThermoFisher Evolution 220); while μ’_s_ was determined via Mie theory. In half of the phantoms only the scattering agent was dissolved, while the remaining half had both scattering and absorbing agents. Therefore, six different scattering agent concentrations were used on all phantoms: μ’_s_ was between 2.15 and 21 cm^-1^ from 450 to 800 nm, while a fixed concentration of the absorbing agent was used on half of the phantoms in order to have a μ_a_ span between 0 and 10 cm^-1^ at the same wavelength range indicated previously [Fig. S2(a, b)].


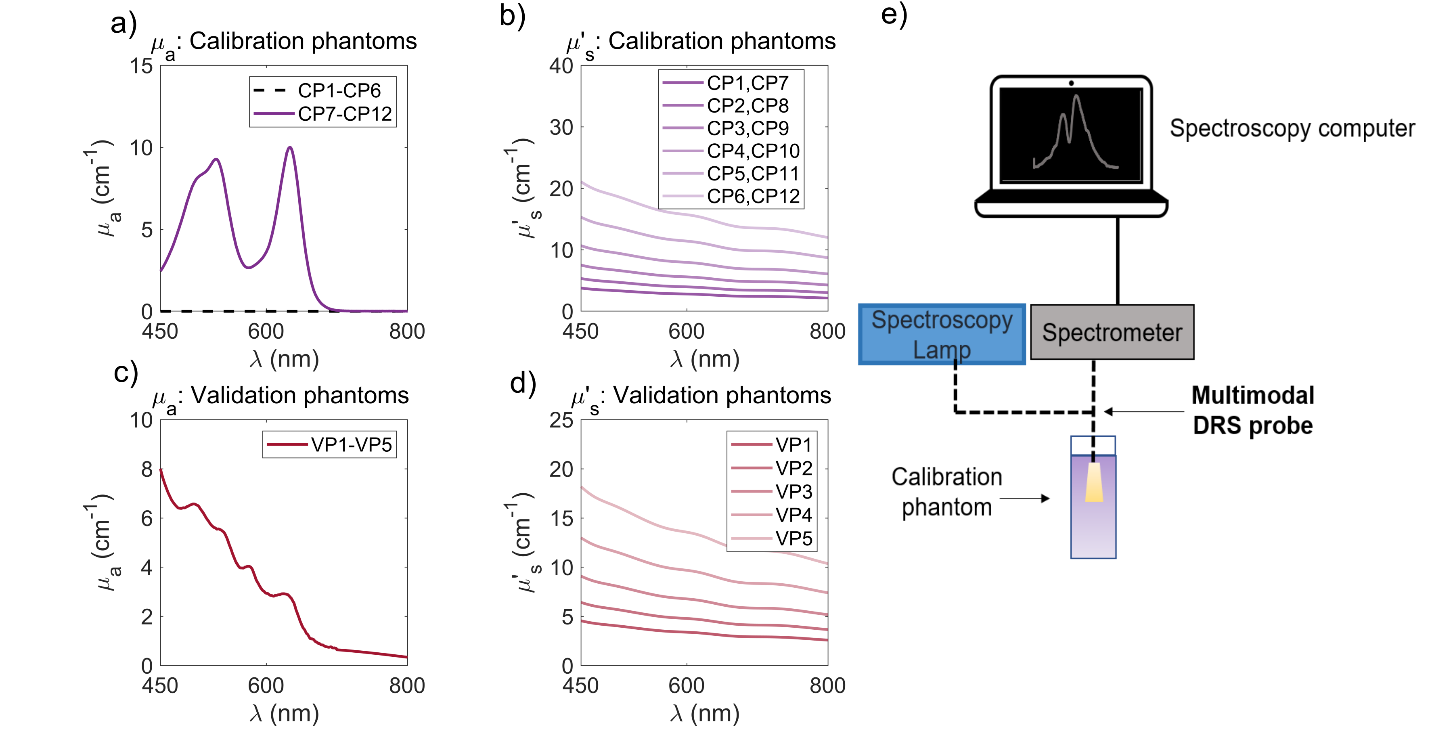


**Fig. S2.** Calibration and validation phantoms optical properties, and system configuration for DRS calibration. calibration phantoms made with polystyrene beads and food dye: μ_a_ and μ’_s_ ranged between 0-10 cm^-1^ (a). and 2.15-21 cm^-1^(b) between 450-800 nm, respectively. Validation phantoms made with polystyrene beads and bovine hemoglobin: μ_a_ and μ’_s_ ranged between 0-8 cm^-1^ (c). and 2.59-18.18 cm^-1^(d) between 450-800 nm, respectively. e) Configuration used for the DRS calibration and validation steps.

To calibrate for the daily variations in light intensity, reflectance from a 5% reflectance standard (SRS-05-10, Labsphere, NH, US) was collected after acquiring raw intensity from the phantoms. Raw intensity was thus converted to absolute reflectance using the equation:

$\text{R(λ)=}\frac{\text{I(λ)}_{\text{sample}}\text{-}\text{I(λ)}_{\text{background}}}{\left( \text{I(λ)}_{\text{standard}}\text{-}\text{I(λ)}_{\text{background}} \right)\text{×20}}$ (S1)

Where $\text{R}\text{(}\text{λ}\text{)}$ is the absolute diffuse reflectance,${\text{I}\text{(}\text{λ}\text{)}}_{\text{sample}}$ is the raw intensity, ${\text{I}\text{(}\text{λ}\text{)}}_{\text{background}}$ is the background noise, ${\text{I}\text{(}\text{λ}\text{)}}_{\text{standard}}$ is the intensity from the calibration standard and 20 converts the 5% reflectance level of the standard to a 100% value. With the DRS configuration previously described the distal end (tip) of the probe was placed in contact with the liquid phantom and raw intensity was collected at an integration time of 70 ms using custom LabVIEW software (National Instruments, USA).

The known optical properties of the phantoms were then correlated with the measured reflectance to create the LUT [Fig. S3(a)].


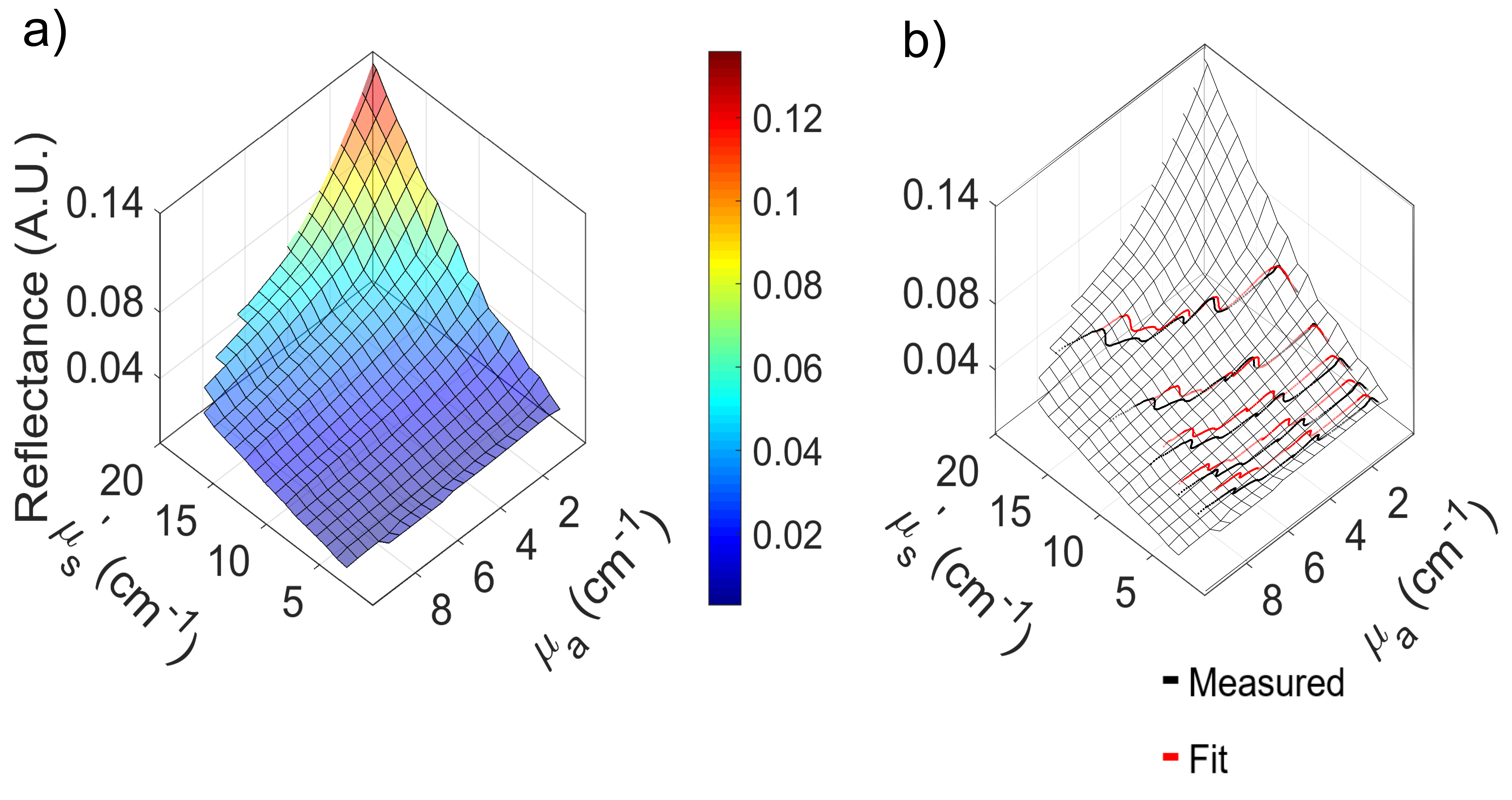


**Fig. S3.** a) LUT constructed by interpolation between the raw phantom reflectances. b) Overlay of the validation phantoms measured reflectance (black lines), the fit produced by the LUT (red lines) and the LUT (grid). Validation phantom properties ranged from 2.59- 18.18 cm^-1^ and 0-8 cm^-1^ from 450 to 800 nm for μ’_s_ and μ_a_ respectively.

***DRS Validation***

To test the accuracy of the model, 5 validation phantoms were created using the same scattering agent previously described (polystyrene beads) but bovine hemoglobin (H2625, Millipore Sigma) was used as absorbing agent as it represented more closely the expected absorption in animal tissue, and also would provide the response of the LUT do a different absorber than the one used for its construction. The determination of μ_a_ and μ’_s_ for the validation phantoms and the collection of reflectance was identical to the calibration phantom set. However, the ranges for both μ_a_ and μ’_s_ were chosen such that they were not the same as in the previous phantom set. Specifically, μ_a_ and μ’_s_ ranges from 450 to 800 nm were 0-8 and 2.59-18.18 cm^-1^, respectively [Fig. S1(c, d)].

The constraining equations used to extract optical properties were:

$\text{µ}_{\text{af}}\left( \text{λ} \right)\text{=}\left[ \text{μ}_{\text{a}}\left( \text{λ} \right)\text{×}\text{C}_{\text{BvHb}} \right]$ (S2)

${\text{μ}^{\text{'}}}_{\text{s}}\text{(λ)=}{\text{μ}^{\text{'}}}_{\text{s}}\text{(}\text{λ}_{\text{0}}\text{)×}\left( \frac{\text{λ}}{\text{λ}_{\text{r}}} \right)^{\text{-B}}$ (S3)

For (S2), $\text{µ}_{\text{af}}\left( \text{λ} \right)$ is the final absorption coefficient at each wavelength, $\text{μ}_{\text{a}}\left( \text{λ} \right)$ is the absorption coefficient determined by the Beer-Lambert law for the absorber, and $\text{C}_{\text{Bv}\text{Hb}}$ is the percent of bovine hemoglobin in the sample. For (S3), ${\text{μ}^{\text{'}}}_{\text{s}}\text{(λ)}$ is the reduced scattering coefficient, ${\text{μ}^{\text{'}}}_{\text{s}}\text{(}\text{λ}_{\text{0}}\text{)}$ is the reference reduced scattering coefficient at 630 nm,$\text{λ}$ is all wavelengths, *B* is the scattering exponent which characterizes the wavelength dependence of ${\text{μ}^{\text{'}}}_{\text{s}}$, and $\text{λ}_{\text{r}}$ is the reference wavelength (685 nm).

As the measured reflectance and previously known optical properties in the validation phantoms fall within the range of values specified by the LUT, a nonlinear optimization routine (NLOR) implemented in Matlab (MathWorks, MA, USA), was used where the measured reflectance per wavelength for each validation phantom is provided to the inverse-LUT model to estimate a reflectance and extract the optical properties (μ_a_ and μ’_s_) accordingly. Boundary conditions for the NLOR are listed in Table S2.

**Table S2**

**Boundary conditions for the Validation step**

| Variable | Boundary conditions |
| --- | --- |
| $\text{C}_{\text{BvHb}}$ (%) | 0-100 |
| ${\text{μ}^{\text{'}}}_{\text{s}}\text{(}\text{λ}_{\text{0}}\text{)}$(cm^-1^) | 2.8-12.0 |
| *B* | 0-4 |

The threshold for goodness of fit was determined by minimizing the sum of the chi-square distance (χ^2^) between the LUT-based and measured reflectance [Fig. S3(b)]. Average errors for μ_a_ and μ’_s_ were determined for all validation phantoms.

***Sampling Depth***

The highest scattering and absorbing calibration phantom (CP12) was placed in a 5 mL beaker that had a highly absorbing phantom layer (μ_a_>100 cm^-1^) on the bottom, made of poly-dimethylsiloxane (PDMS) and black ink, which was assumed to attenuate any photons incident to it. Next, using the previously described configuration for DRS acquisition [Fig.S2(e)], the tip of the probe was placed in contact with the highly absorbing phantom layer and lifted in 10 μm increments from 0 to 500 μm using a micrometer scale. At each position, DRS data was collected with an integration time of 70 ms, thus enabling to create a correlation between the lifted distance and the measured spectra.

Reflectance was calculated using Eq.1 for each spectra, and the values at 542 nm were extracted from all collected data, followed by interpolation to correlate the obtained reflectance values with the distance increments. From [Fig. S4(a)] it can be seen that the reflectance increases with the distance until it starts to reach a plateau around 450 μm; sampling depth was then defined as the depth reached by 50% of the photons. Therefore, the measured sampling depth was determined to be 263±7 μm [Fig. S4(a)]. Finally, the process was repeated for the sampling depths across all wavelengths and the relationship between sampling depth and wavelength was obtained [Fig. S4(b)].


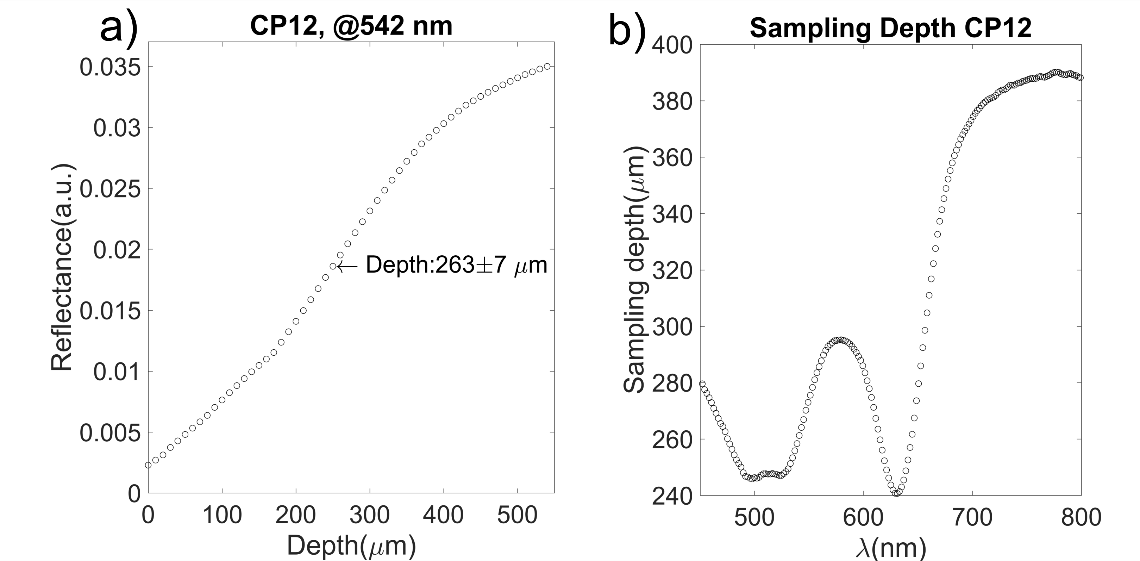


**Fig. S4.** Sampling depth for the DRS configuration of the multimodal probe. a) Correlation between reflectance and the distance increment. The probe was lifted in 10 μm from 0 to 500 μm and DRS was acquired at each distance. Reflectance at 542 nm was extracted at each position and an interpolation was performed to complete the trend of the data. Sampling depth was calculated as the distance where 50% of the photons reach the detector. b) Sampling depth at every wavelength was determined by repeating the procedure for a) per wavelength and correlating the distance with each wavelength value.

***Ex vivo changes in reflectance with positive or negative displacement***

Changes in reflectance due to positive displacement between the tip of the probe indicated that the reflectance remained without significant change for the first 300 μm, it increased between 400-500 μm and decreased between 600-1000 μm [Fig. S5(a)]. For negative displacement, the most distinguishable changes were in the 500-600 nm spectral region were seen, where the Q bands of hemoglobin exhibited a progressive change from oxy-hemoglobin to deoxy-hemoglobin [Fig. S5(b)].


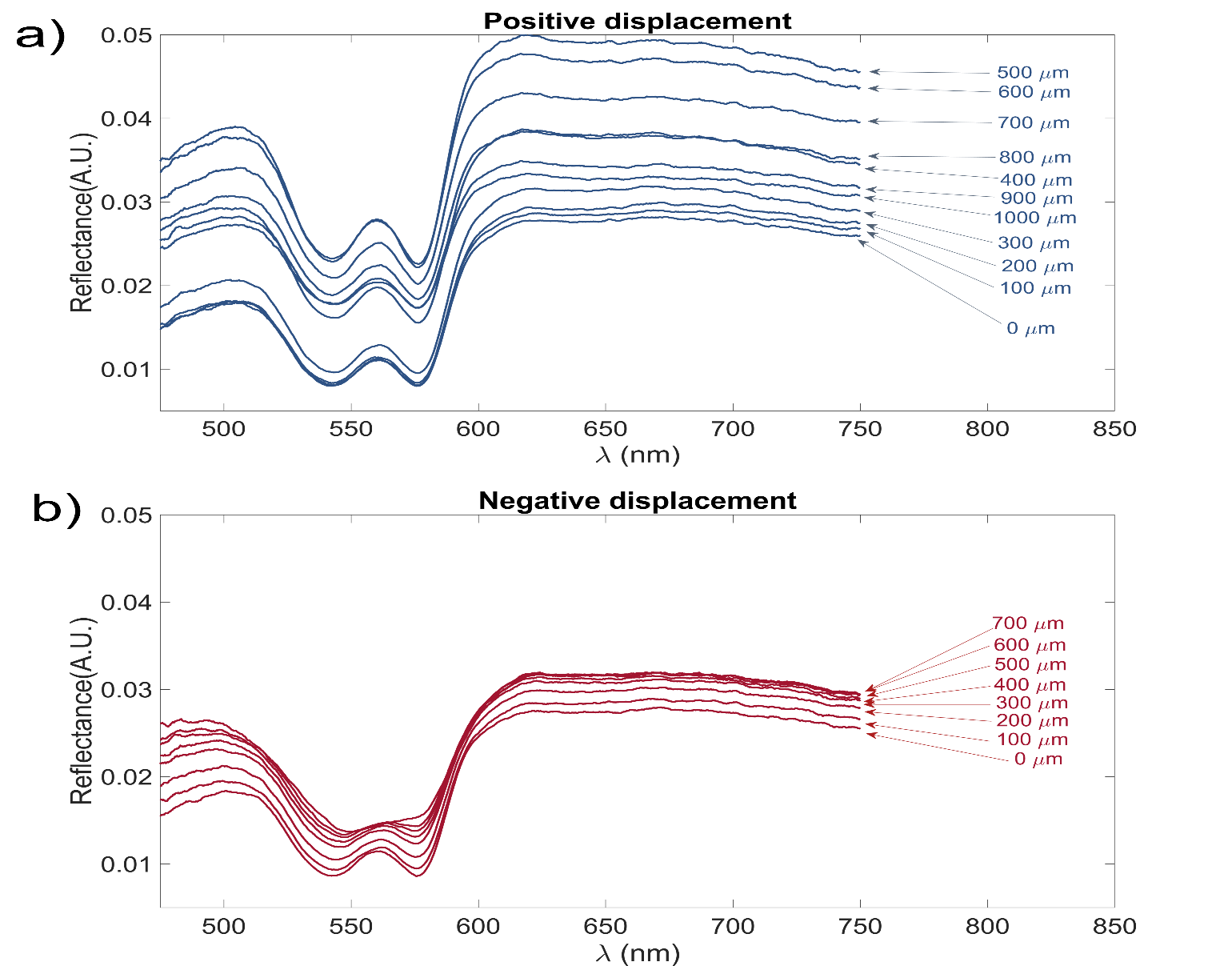


**Fig. S5.** *Ex vivo* changes in reflectance. a) Changes with positive displacement. Reflectance changes minimally between 0-300μm, increases between 100-500 μm and decreases between 600-1000 μm. b) Changes in positive displacement. Reflectance increments minimally but the changes in the Qbands of hemoglobin (500-600 nm range) show the transition from oxy to deoxy-hemoglobin.

***Qratios from ex vivo spectra***

The Qratio was determined ex vivo from 2 tumors of different mice at each angle (0,5,15,30°) and at 100μm distance increments (Fig. S6). This analysis demonstrated that the Qratio changed with angular/positional variations providing the rationale for its extraction from *in vivo* optimal data.


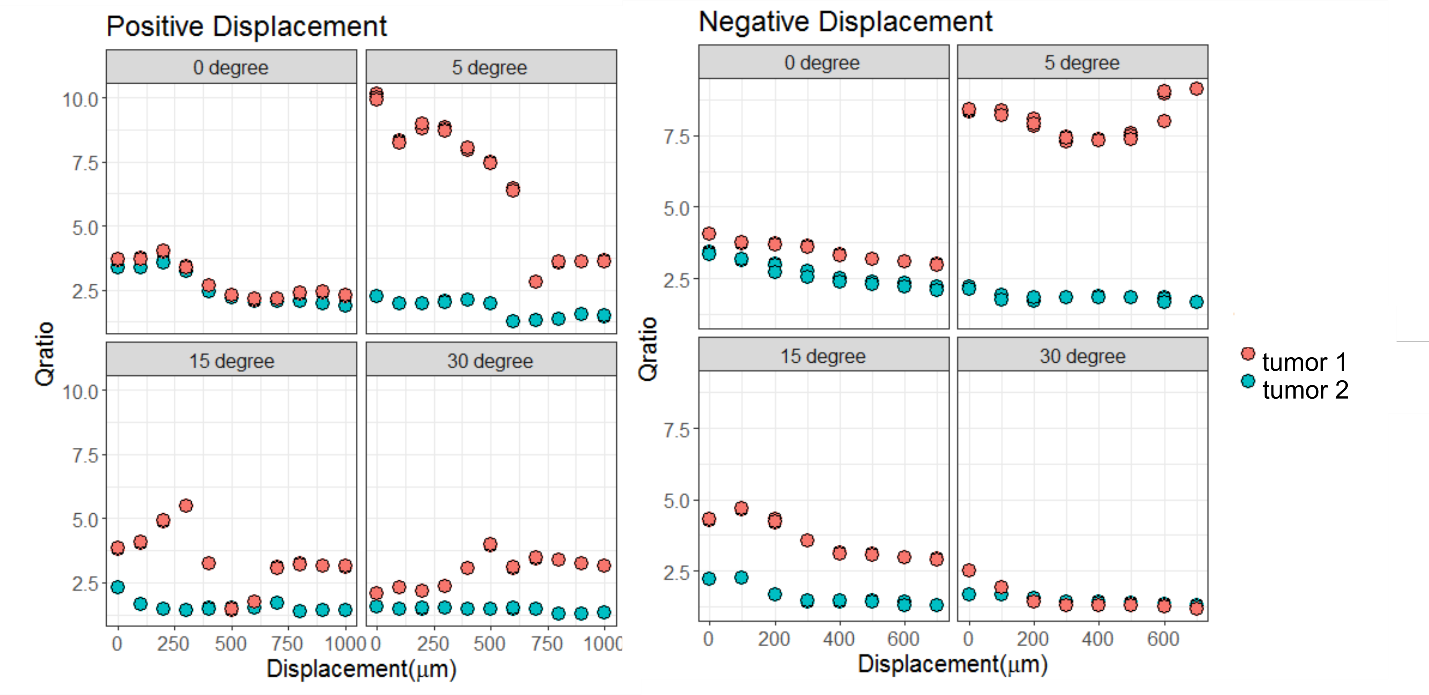


**Fig. S6.** Ex vivo Qratios determined at each angular position (0,5,15,30°) for positive displacement (left panels) and negative displacement (right panels).

***Filtering Algorithm***

A filtering algorithm was constructed to filter data that had angle or displacement (positive or negative) artifacts to avoid biasing the DRS data to be analyzed: First, reflectance would be discarded if it was above 0.05 or below 0.01 at 475 nm. The value for 0.01 was selected as a lower limit as it would correspond to reflectance outside the range quantified by the LUT. Later, the Qratio would be calculated for the spectra that passed the first filter and if the value was outside ±1SD of the previously calculated mean in vivo value (<1.99 and above >3.09) it would be discarded. Finally, a confidence interval was created for the remaining spectra by the determining mean and SD across all wavelengths. Spectra that were outside the mean ±1SD region were discarded. The flowchart is summarized in Fig. S7.


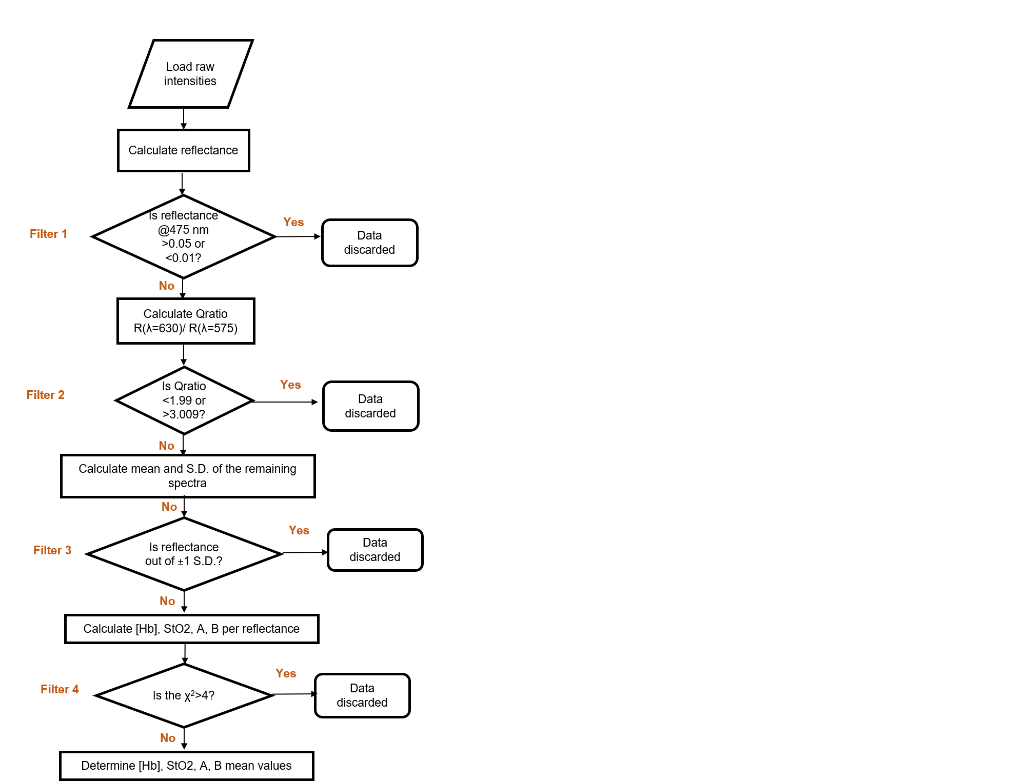


**Fig. S7.** Filtering algorithm for the *in vivo* acquired DRS data. Filtering was performed to eliminate spectra with high values due to positive displacement (Filter 1), negative displacement as assessed by the *in vivo* calculated Q ratio (Filter 2) and a ±1SD confidence interval for the remaining spectra (Filter 3). Mean physiological values were calculated from spectra for which the difference between the measured reflectance and the fit would have χ^2^<4.

***DRS post-processing (Inverse LUT model)***

Optical properties and physiological values from tissue (StO_2_, tHb, HbO_2_, Hb) were quantified using the inverse LUT model between 475-685 nm using the same NLOR described for the validation phantoms. The constraining equation for scattering remained the same as for the validation phantoms (S3). For absorption, assuming hemoglobin is the only absorber, the constraining equation for the absorption coefficient is written as reported elsewhere:

$\text{µ}_{\text{a}}\text{(λ)=[}\text{tHb}\text{]×[}\text{α}\varepsilon_{\text{HbO}_{\text{2}}}\text{(λ)+(1-}\text{α}\text{)}\varepsilon_{\text{Hb}_{\text{2}}}\text{(λ)}$] (S4)

Where [tHb] is the total hemoglobin content (in mg/mL), α is the oxygen saturation (StO_2_, %), $\varepsilon_{{HbO}_{2}}$ and $\varepsilon_{{Hb}_{2}}$ are the extinction coefficients for oxy-hemoglobin and deoxyhemoglobin (in mL/mg·cm), respectively. Boundary conditions for the *in vivo* DRS analysis are listed in Table S3.

Table S3

Boundary conditions for *in vivo* mouse colon spectroscopy

| Variable | Boundary conditions |
| --- | --- |
| ${StO}_{2}$ (%) | 0-100 |
| ${\text{μ}^{\text{'}}}_{\text{s}}\text{(}\text{λ}_{\text{0}}\text{)}$(cm^-1^) | 2.15-21.0 |
| *B* | 0.0-4.0 |
| tHb(mg/mL) | 0-150 |

The NLOR performed up to 6×10^3^ iterations and a 4^th^ and final filter was used, where the χ^2^ distance across all wavelengths was calculated between the LUT-based reflectance and the measured reflectance. Data was discarded if χ^2^>4, a value that was selected as it allowed the acceptance of 77% of the DRS data (data not shown).

***DRS-derived physiological information***

DRS-derived physiological information per mouse per group is displayed in Fig. S8.


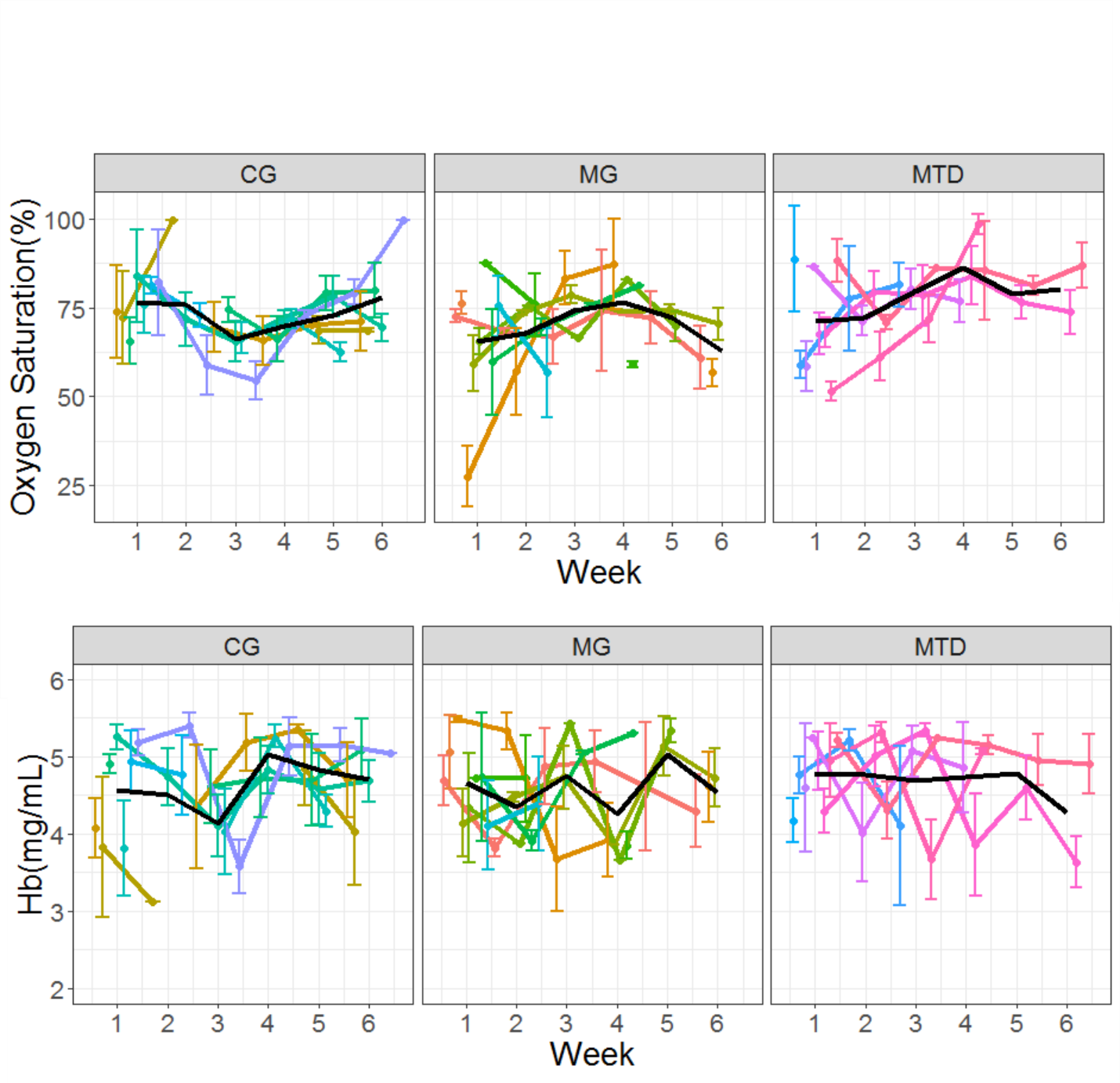


**Fig. S8.** Longitudinal DRS-derived physiological data. Upper panel: Changes in StO_2_Changes in tHb. For both panels, each point of the same color represents the average value (StO_2_ or THb) extracted from the inverse-LUT model from the filtered DRS spectra. Lines of the same color connect measurements of the same animal. Error bars correspond to ±1SD. Black lines are mean values per group.
